# Kernel-Transformed Functional Connectivity Entropy Reveals Network Dedifferentiation in Bipolar Disorder

**DOI:** 10.3390/brainsci16020208

**Published:** 2026-02-10

**Authors:** Nan Zhang, Weichao An, Shengnan Li, Jinglong Wu

**Affiliations:** 1 Faculty of Biomedical Engineering, Shenzhen University of Advanced Technology, Shenzhen 518107, China; 2 Graduate School of Interdisciplinary Science and Engineering in Health Systems, Okayama University, Okayama 700-8530, Japan; 3 International Joint Laboratory of Behavior and Cognitive Science, Zhengzhou Normal University, Zhengzhou 450044, China

**Keywords:** bipolar disorder, resting-state fMRI, kernel-transformed functional connectivity, functional connectivity entropy, brain network dysregulation

## Abstract

**Highlights:**

**What are the main findings?**

- Developed a kernel-transformed functional connectivity (FC) entropy framework that acts as a reweighting filter to enhance connectivity contrast and suppress noise, facilitating sensitive quantification of weight distributions.
- Bipolar Disorder (BD) exhibited widespread network dedifferentiation (increased entropy) at global, modular (DMN, VAN, DAN), and nodal levels, independent of head motion effects.

**What are the implications of the main findings?**

- Kernel-transformed FC entropy provides a distribution-sensitive complement to conventional linear FC metrics for quantifying network dysregulation in BD.
- Multiscale entropy abnormalities support kernel-transformed entropy as a promising candidate metric for characterizing symptom-related networks and potential stratification in BD.

**Abstract:**

**Background**: Resting-state functional MRI (rs-fMRI) studies typically rely on linear Pearson correlation to characterize brain connectivity, potentially overlooking the distributional characteristics of functional networks. This study introduces a kernel-transformed functional connectivity (FC) entropy framework to quantify network dedifferentiation in bipolar disorder (BD). **Methods**: We utilized a Gaussian kernel function to execute a nonlinear similarity transformation (referred to as reweighting) on standard linear correlation matrices. This approach acts as a functional filter to amplify the contrast between strong and weak connections. Multiscale entropy (global, modular, and nodal) was subsequently calculated to characterize the uniformity of connectivity weight distributions. **Results**: Compared to Normal Controls (NCs), patients with BD exhibited significantly higher entropy at the global level and within the Default Mode, Salience, and Somatosensory-Motor networks, indicating widespread network dedifferentiation (distributional flattening). These alterations were robust across different kernel widths and remained significant after rigorously controlling for head motion (Mean FD). Furthermore, manic symptom severity (YMRS) was negatively correlated with global entropy, suggesting a pathological “locking-in” or rigidity of specific neural circuits during manic states. **Conclusions**: The kernel-transformed FC entropy serves as a distribution-sensitive complement to conventional linear metrics. Our findings highlight network dedifferentiation as a key pathophysiological feature of BD and suggest this framework as a promising candidate metric for characterizing network dysregulation.

## 1. Introduction

Bipolar disorder (BD) is a psychiatric illness characterized by extreme mood fluctuations, including recurrent manic and depressive episodes, which severely impair cognition and behavior [1,2,3]. Manic episodes are typically associated with increased energy, heightened impulsivity, and impaired judgment, whereas depressive episodes manifest as low energy, sadness, and cognitive deficits. Accumulating evidence indicates that these clinical features are linked to dysfunction in functional brain networks involved in emotion regulation, cognition, and decision-making [4,5]. Previous studies have reported disruptions within- and between-network connectivity in the default mode network (DMN), ventral attention network (VAN), and dorsal attention network (DAN), which may contribute to the affective cognitive impairment observed in BD [6,7,8]. Therefore, a key challenge is to capture these BD-related abnormalities in functional organization using quantitative metrics that can reflect the holistic topological properties of the brain network.

Resting-state functional magnetic resonance imaging (rs-fMRI) provides a robust noninvasive modality to probe spontaneous brain activity [9]. Functional connectivity (FC) analysis based on rs-fMRI, typically estimated via Pearson correlation between time series, has been widely deployed to characterize brain network organization and its reconfiguration in psychiatric disorders [10,11]. While these conventional metrics effectively quantify the linear strength of pairwise coupling, relying solely on individual edge strengths may fail to capture the intricate distributional properties inherent in complex neural systems [12]. For instance, subtle deviations in global topology—such as the interplay between integration and segregation—might be masked when connectivity is analyzed exclusively through mean correlation strength, disregarding the diversity and dispersion of the connectivity weight profile [13,14].

To achieve a more comprehensive characterization of network organization, entropy-based measures have been introduced to quantify the uniformity of connectivity configurations [15,16,17]. In the context of weighted brain networks, Shannon entropy serves as a robust metric for the uniformity of the weight distribution [18]. A highly organized brain network typically exhibits a distinctly skewed distribution of connection weights (i.e., low entropy), thereby maintaining a clear demarcation between strong intramodular connections and weak intermodular links [19]. Conversely, elevated entropy implies a more uniform or flattened distribution of connectivity weights. In psychiatric conditions, such entropic increases may reflect a state of “network dedifferentiation” or a loss of functional specialization, where the boundaries between functional modules become blurred, and the informational distinctiveness of network processing is compromised [20,21,22].

However, applying entropy measures directly to raw linear correlation matrices can be constrained by the inherently dense and noise-prone nature of fMRI connectivity data [23]. The intrinsic contrast between strong and weak connections in raw Pearson matrices is often attenuated, potentially masking subtle distributional shifts. To address this limitation, this study proposes an analysis framework termed kernel-transformed functional connectivity entropy. Distinct from attempting to estimate nonlinear statistical dependencies, this approach employs a Gaussian kernel function to perform a nonlinear similarity transformation on the standard linear correlation matrix [24,25]. This transformation acts as a nonlinear reweighting filter that expands the contrast of the connectivity profile: it enhances the separation between high-affinity and low-affinity connections, thereby making the entropy metric more sensitive to detecting pathological alterations in network uniformity and dedifferentiation.

Building on this framework, we investigated the multiscale organization of brain networks in BD across global, modular, and nodal levels. We hypothesized that BD patients would exhibit elevated entropy compared to normal controls (NCs), reflecting a widespread dedifferentiation of functional networks (i.e., increased uniformity of connection weights). Additionally, we conducted exploratory analyses to evaluate the relationships between kernel-transformed FC entropy and clinical symptom severity (YMRS and HAMD). In summary, this study aims to validate kernel-transformed entropy as a sensitive and robust distributional marker for characterizing the network-level pathophysiology of BD.

## 2. Materials and Methods

### 2.1. Participants

The dataset utilized in this study was retrieved from the publicly available UCLA Consortium for Neuropsychiatric Phenomics LA5c Study (OpenfMRI database, ds000030) [26]. A total of 90 participants were enrolled and divided into two groups: individuals diagnosed with bipolar disorder (BD) and age- and sex-matched normal controls (NCs). Diagnoses followed the Diagnostic and Statistical Manual of Mental Disorders and were based on the Structured Clinical Interview for DSM-IV (SCID-I). Statistical analyses confirmed that the groups were well-balanced, with no significant between-group differences in age or sex distribution.

The BD group comprised 45 patients (mean age = 35.53 ± 9.21 years; 26 males). All patients underwent clinical assessments using the Young Mania Rating Scale (YMRS) and the 17-item Hamilton Depression Rating Scale (HAMD) [27,28] to quantify the severity of manic and depressive symptoms, respectively, and to provide a comprehensive characterization of their clinical status. The NC group consisted of 45 participants (mean age = 35.56 ± 9.18 years; 26 males). Secondary analysis of this dataset was approved by the UCLA Institutional Review Board, and all procedures were conducted in accordance with the relevant ethical guidelines and regulations. A detailed summary of the demographic and clinical characteristics is presented in Table 1.

### 2.2. Data Acquisition and Data Processing

All functional MRI (fMRI) data were acquired on a 3.0-Tesla Siemens Trio scanner (Siemens, Erlangen, Germany) using a T2*-weighted echo-planar imaging (EPI) sequence. The acquisition parameters were as follows: slice thickness = 4.0 mm; 34 slices; repetition time (TR) = 2000 ms; echo time (TE) = 30 ms; flip angle = 90°; matrix size = 64 × 64; field of view (FOV) = 192 mm; and 152 volumes (time points).

Preprocessing of the rs-fMRI data was performed using Data Processing and Analysis for (Resting-State) Brain Imaging (DPABI) (v9.0, The R-fMRI Lab, Institute of Psychology, CAS, Beijing, China) based on SPM25 (Wellcome Trust Centre for Neuroimaging, London, UK) [29]. The first 10 volumes were discarded to allow for magnetization equilibrium and participant adaptation. Slice-timing correction was performed to account for inter-slice acquisition delays, followed by head-motion correction using rigid-body realignment. After realignment, functional images were spatially normalized to the Montreal Neurological Institute (MNI) standard space and resampled to 3 × 3 × 3 mm^3^ isotropic voxels. The normalized images were then spatially smoothed with a Gaussian kernel of 6 mm full width at half-maximum (FWHM).

To preclude the reintroduction of noise-related frequency components, nuisance covariate regression was performed prior to temporal filtering. Linear trends were removed, and nuisance covariates were regressed out from the time series, including the global signal, mean signals from cerebrospinal fluid (CSF) and white matter (WM), and head-motion effects modeled using the Friston 24-parameter model. Given that entropy metrics are highly sensitive to the overall distributional shift in connectivity weights, GSR was explicitly applied to minimize widespread physiological noise (e.g., respiration and cardiac activity) that could artificially inflate whole-brain synchronization. This step ensures that the entropy measures reflect the intrinsic topological organization of the network rather than global noise levels [25]. Finally, temporal band-pass filtering (0.01–0.08 Hz) was applied to the residual time series. The resulting denoised rs-fMRI data were used for subsequent analyses.

To rigorously mitigate the influence of head motion, the mean Framewise Displacement (FD) was calculated for each participant using Jenkinson’s formula. Statistical analysis demonstrated no significant differences in mean FD between the BD and NC groups (*p* = 0.191; Table 1), indicating that the groups were well-matched with respect to head motion. Despite this matching, mean FD was further included as a nuisance covariate in all subsequent group-level statistical analyses to minimize any potential confounding effects.

### 2.3. Functional Connectivity Construction and Kernelization

After preprocessing, the brain was partitioned into 264 regions of interest (ROIs) based on the Power atlas [30]. The Power atlas is a widely used, functionally defined parcellation comprising 264 ROIs distributed across the cerebral cortex, subcortex, and cerebellum. Each ROI is assigned to one of 12 functional modules, including motor and somatosensory (MSN), cingulo-opercular (CON), auditory (AUD), default mode (DMN), visual (VIS), fronto-parietal (FPN), salience (SAL), subcortical (SUB), ventral attention (VAN), and dorsal attention (DAN), as well as two modules labeled as undefined. In this study, multiscale topological analyses were performed at the global, modular, and nodal levels. For modular-level analyses, only ROIs belonging to the 10 defined modules were retained, whereas ROIs labeled as undefined were excluded.

For each participant, a mean BOLD time series was extracted for each ROI by averaging the signals across all voxels within the ROI. Pairwise functional connectivity (FC) was subsequently quantified as the Pearson correlation coefficient [31] between the time series of every pair of ROIs, yielding an individual 264 × 264 correlation matrix $R=[r_{ij}]$:

(1) $$r\left(x_{i},x_{j}\right)=\frac{\sum_{t=1}^{n}\left(x_{it}-\bar{x_{i}})(x_{jt}-\bar{x_{j}}\right)}{\sqrt{\sum_{t=1}^{n}{\left(x_{it}-\bar{x_{i}}\right)}^{2}}\sqrt{\sum_{t=1}^{n}{\left(x_{jt}-\bar{x_{j}}\right)}^{2}}}$$

where $x_{i}=\left[x_{i1},\ldots {,x}_{in}\right]$ and $x_{j}=\left[x_{j1},\ldots {,x}_{jn}\right]$ denote the ROI time series for regions i and j, respectively, n is the number of time points, and $\bar{x_{i}}$ and $\bar{x_{j}}$ are the means.

To characterize FC similarity and construct an affinity network, correlation values were transformed into nonnegative edge weights by kernelization using a Gaussian kernel [32]:

(2) $$w_{ij}=exp(-\frac{1-r(x_{i},x_{j})}{\sigma^{2}})$$

where $W=[w_{ij}]$ is the kernel-transformed affinity matrix and σ is the kernel width parameter. It is important to note that this transformation is applied to linear correlation coefficients. Thus, Equation (2) represents a nonlinear reweighting of linear connectivity strengths, designed to expand the contrast between strong and weak connections and enhance the sensitivity to the weight distribution, rather than a direct estimation of nonlinear statistical dependencies.

To evaluate robustness of the kernel setting, all subsequent analyses were performed separately with σ∈{0.3,0.5,0.7}. The parameter σ = 0.5 was selected as the primary setting, as it empirically achieves an optimal balance between network sparsity and smoothness: a smaller *σ* (e.g., 0.3) tends to excessively penalize moderate connections, whereas a larger *σ* (e.g., 0.7) may over-smooth the weight distribution, thereby diminishing the discriminative power of the entropy metric. Notably, Fisher’s r-to-z transformation was omitted in this framework. This is because the kernel mapping naturally constrains the weights within the [0, 1] interval, and the subsequent entropy analysis targets the distributional uniformity of these weights rather than parametric inference based on raw correlation coefficients.

### 2.4. Entropy Estimation

We quantified the uniformity of the kernel-transformed similarity weights using Shannon entropy. For a given set of edges E, kernel weights were converted into a discrete probability mass function by normalizing the weights:

(3) $$p_{ij}=\frac{w_{ij}}{\sum_{(u,v)\in E,}w_{uv}},\;\;(i,j)\in E$$

Shannon entropy [33] was then computed as

(4) $$H\left(E\right)=-\sum_{(i,j)\in E}p_{ij}\log_{2}p_{ij}$$

Entropy was evaluated at three levels:

At the global level, $E_{g}=\left\{\left(i,j\right)|1\leq i<j\leq 264\right\}$ included all unique edges (upper triangle, excluding the diagonal) of the 264 × 264 affinity matrix, and global entropy was computed as $H_{g}=H\left(E_{g}\right)$.

At the nodal level, for each ROI i, $E_{i}=\left\{\left(i,j\right)|1\leq j\leq 264,\;j\;\neq i\right\}$ contained all edges incident to node i, and node entropy was computed as $H_{i}=H\left(E_{i}\right)$, reflecting how evenly the similarity weights from ROI i are distributed across its connections.

At the modular level, ROIs were grouped according to the labels in the Power atlas. We included all defined modules (10 in total) and excluded ROIs labeled as undefined from module-level analyses. For each module m with ROI set $V_{m}$, within-module edges were defined as $E_{m}=\;\left\{\left(u,v\right)|u<v,\;u\in V_{m},\;v\;{\in V}_{m}\right\}$, and module entropy was computed as $H_{m}=H\left(E_{m}\right)$, characterizing the dispersion of similarity weights within each functional module.

All entropy measures were computed separately for σ = 0.3, 0.5, and 0.7, and were subsequently used for group-level comparisons between BD and NCs. We treated *σ* = 0.5 as the primary setting for reporting, while σ = 0.3 and σ = 0.7 were used for sensitivity analyses.

In this framework, higher entropy indicates a more uniform distribution of connectivity weights, indicating a lack of distinctiveness between strong and weak connections. This state is biologically interpreted as network dedifferentiation or a loss of functional segregation.

### 2.5. Statistical Analysis

All statistical analyses were conducted using SPSS 20.0 (IBM Corp., Armonk, NY, USA) [34]. Demographic comparisons between the two groups were performed using independent-samples *t*-tests for age and Pearson’s χ^2^ test for sex distribution, with the significance threshold set at *p* < 0.05. To evaluate between-group differences (BD vs. NC) in entropy measures across global, nodal, and modular levels, Analysis of Covariance (ANCOVA) was employed. Age, sex, and mean FD were included as covariates in the model. To control multiple comparisons, the False Discovery Rate (FDR) correction was applied separately at the nodal level and at the modular level, with an FDR-adjusted significance threshold of *p* < 0.05. For global entropy, significant results were further verified using Bonferroni correction for the three kernel width settings.

For clinical correlations, exploratory partial correlation analyses were conducted to evaluate the associations between entropy measures and symptom severity (YMRS and HAMD) in the BD group, while accounting for age, sex, and mean FD as covariates. Given the exploratory nature of these analyses and the limited sample size, these correlations were reported at an uncorrected significance threshold of *p* < 0.05 to identify potential trends for future investigation.

## 3. Results

### 3.1. Demographic and Clinical Characteristics

Demographic and clinical characteristics are summarized in Table 1. Independent-samples *t*-tests revealed no significant between-group differences in age (*p* = 0.991) or mean FD (*p* = 0.191). Furthermore, Pearson’s χ^2^ test indicated that the sex distribution was perfectly balanced between the two groups (*p* = 1.000). These results confirm that the BD and NC groups were well-matched in terms of head motion, minimizing the potential for motion-related artifacts. Clinical symptom severity was further evaluated in the BD group using YMRS (mean ± SD: 10.49 ± 11.22, range: 0–37) and the 17-item HAMD (mean ± SD: 11.62 ± 8.43, range: 0–32).

### 3.2. Alteration of Global Entropy in BD Patients

**Figure 1:**
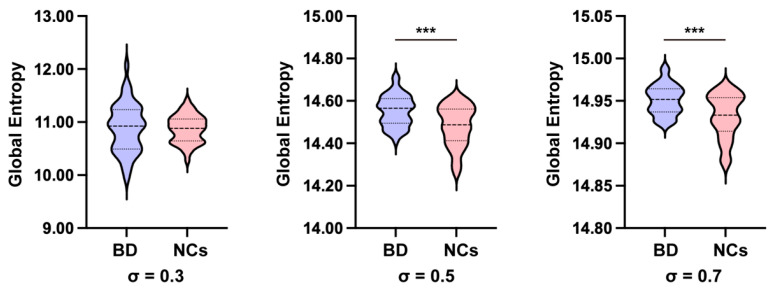
Group differences in global entropy across varying kernel widths. Comparisons were performed using ANCOVA (covariates: age, sex, mean FD). Significant increases in BD were observed at *σ* = 0.5 and *σ* = 0.7 (Bonferroni corrected, *** *p* < 0.001), but not at *σ* = 0.3.

Group comparisons of global entropy were conducted using Analysis of Covariance (ANCOVA), with age, sex, and mean FD included as covariates. As illustrated in Figure 1, the group difference was not significant at *σ* = 0.3 (*F* (1, 85) = 0.023, *p* = 0.879). This lack of significance is likely attributable to the smaller kernel width, which induced excessive sparsity in the affinity matrix, thereby constraining the sensitivity to distributional shifts. In contrast, BD participants exhibited significantly higher global entropy than NCs at *σ* = 0.5 (*F* (1, 85) = 13.982, *p* < 0.001, 95% CI [0.032, 0.104], *η_p_*^2^ = 0.141) and at *σ* = 0.7 (*F* (1, 85) = 15.433, *p* < 0.001, 95% CI [0.009, 0.028], *η_p_*^2^ = 0.154). Notably, these findings remained significant after Bonferroni correction for the three kernel width settings. These results demonstrate that, at empirically optimal kernel scales, BD is characterized by a more uniform distribution of connectivity weights (i.e., higher entropy), indicative of a global dedifferentiation of the functional connectome.

### 3.3. Significant Differences in Modular Entropy Between BD and NCs

**Figure 2:**
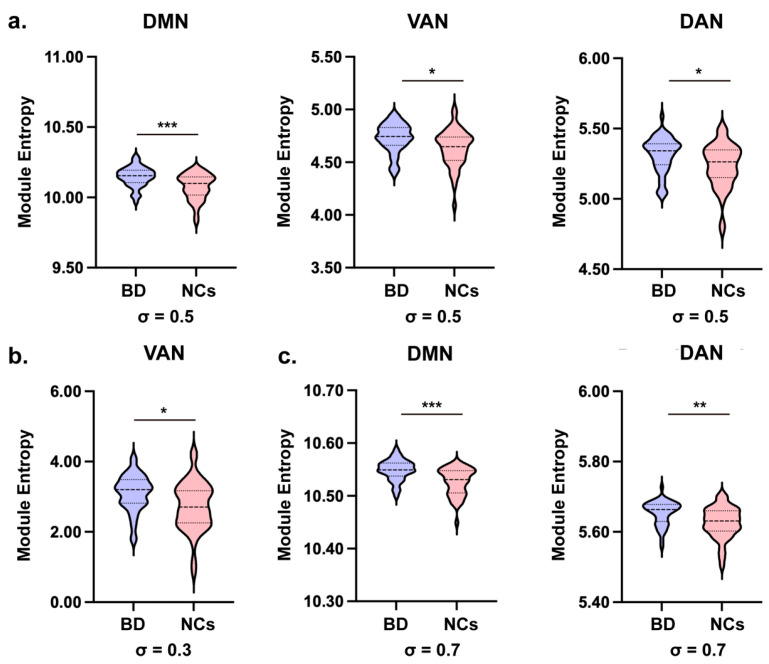
Group comparisons used ANCOVA (covariates: age, sex, mean FD). (**a**) Module entropy comparisons for DMN, VAN, and DAN at scale *σ* = 0.5. (**b**) Module entropy comparison for VAN at scale *σ* = 0.3. (**c**) Module entropy comparisons for DMN and DAN at scale *σ* = 0.7. BD patients showed significantly higher entropy in these networks. Asterisks indicate FDR-corrected significance (* *p* < 0.05, ** *p* < 0.01, *** *p* < 0.001).

Module entropy was calculated for each functional module in the Power-264 atlas. After adjusting for covariates (age, sex, and mean FD) and implementing False Discovery Rate (FDR) correction (*q* < 0.05), significant differences between BD patients and NCs were most prominent at *σ* = 0.5 (Figure 2).

As illustrated in Figure 2a, significantly elevated modular entropy was observed in patients with BD at the primary setting of *σ* = 0.5 across multiple functional systems. Specifically, the BD group exhibited significantly higher entropy within the DMN (*F* (1, 85) = 16.387, *p* < 0.001, *q* = 0.001, 95% CI [0.034, 0.100], *η_p_*^2^ = 0.162), VAN (*F* (1, 85) = 7.738, *p* = 0.007, *q* = 0.024, 95% CI [0.026, 0.158], *η_p_*^2^ = 0.083), and DAN (*F* (1, 85) = 6.389, *p* = 0.013, *q* = 0.037, 95% CI [0.016, 0.138], *η_p_*^2^ = 0.069) modules compared to NCs. Such elevated modular entropy suggests that the internal connectivity within these key cognitive and attentional networks is less segregated and more uniformly distributed in BD patients.

At *σ* = 0.3, as shown in Figure 2b, BD patients showed significantly higher entropy in the VAN (*F* (1, 85) = 9.714, *p* = 0.002, *q* = 0.027, 95% CI [0.144, 0.653], *η_p_*^2^ = 0.102) compared to NCs. Similarly, at *σ* = 0.7, BD patients had significantly higher entropy in the DMN (*F* (1, 85) = 19.644, *p* < 0.001, *q* < 0.001, 95% CI [0.012, 0.031], *η_p_*^2^ = 0.188) and DAN (*F* (1, 85) = 10.977, *p* = 0.001, *q* = 0.007, 95% CI [0.011, 0.045], *η_p_*^2^ = 0.114) modules, as depicted in Figure 2c. However, the VAN module showed only a marginally significant difference (*F* (1, 85) = 5.793, *p* = 0.018, *q* = 0.050, 95% CI [0.003, 0.043], *η_p_*^2^ = 0.064) after FDR correction, indicating a trend toward higher entropy in BD patients.

### 3.4. Different Nodal Entropy of Whole Brain

**Figure 3:**
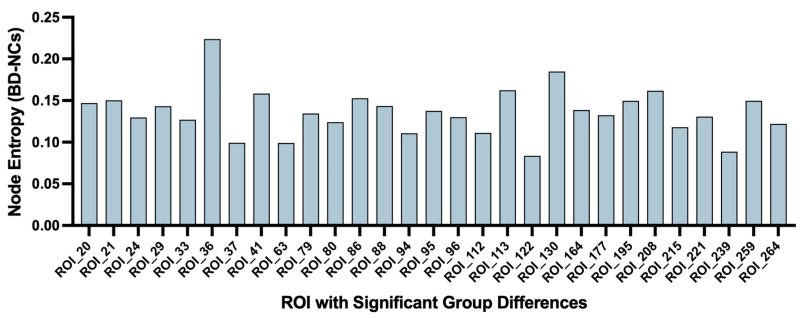
Node entropy differences between BD patients and NCs at *σ* = 0.5. Bar plot showing entropy differences (BD-NCs) for the 29 ROIs with significant alterations. Results were derived from ANCOVA (covariates: age, sex, mean FD) followed by FDR correction (*q* < 0.05). ROI details are listed in Table 2.

Consistent with our multiscale analysis framework, we prioritized *σ* = 0.5 for reporting nodal results, while *σ* = 0.3 and *σ* = 0.7 were utilized for sensitivity analyses. Using ANCOVA with FDR correction (*q* < 0.05) to control for age, sex, and mean FD, we identified 29 ROIs with significantly higher entropy in BD patients compared to NCs (Figure 3).

**Figure 4:**
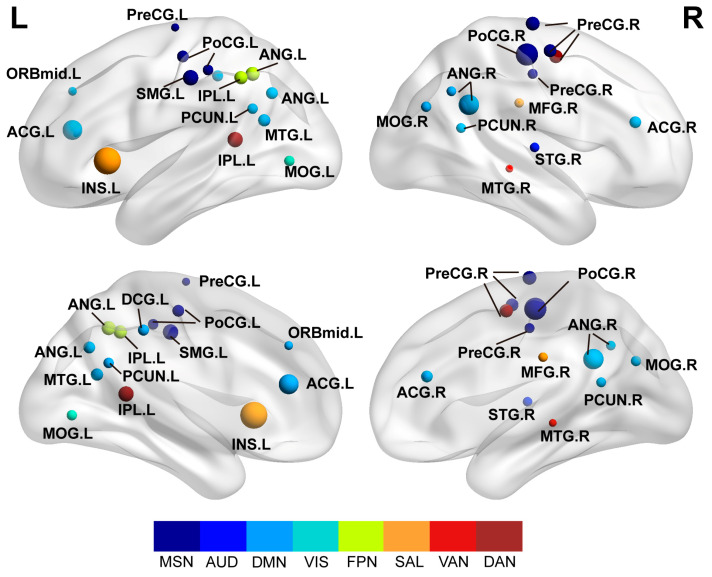
Spatial distribution of significant nodes. The 29 ROIs with significant entropy increases (FDR corrected) are mapped onto the brain surface. Colors represent functional module assignments (e.g., DMN, VAN, DAN).

The bar plot in Figure 3 illustrates the magnitude of these entropy differences. Notably, these aberrant nodes were not randomly distributed but were preferentially located in specific functional modules. As detailed in Table 2, the alterations were clustered in the Default Mode Network (e.g., PCC, ANG), Somatosensory-Motor Network (e.g., PreCG, PoCG), and Attention Networks (e.g., IPL, MTG). The anatomical locations of all differential nodes are shown in Figure 4. This specific nodal distribution aligns with the modular-level findings, providing further evidence for widespread network dedifferentiation in BD.

Due to length constraints, the comprehensive statistical outputs for all nodes across all three kernel coefficients (*σ* = 0.3, 0.5, 0.7) are detailed in the Supplementary Tables S1–S3.

### 3.5. Relationships Between Entropy and Clinical Symptoms

Partial correlation analyses (adjusting for age, sex, and mean FD) were performed to evaluate the associations between entropy measures and clinical scores (YMRS and HAMD). Given the exploratory nature of these analyses and the modest sample size, correlations were reported at an uncorrected significance threshold of *p* < 0.05.

**Figure 5:**
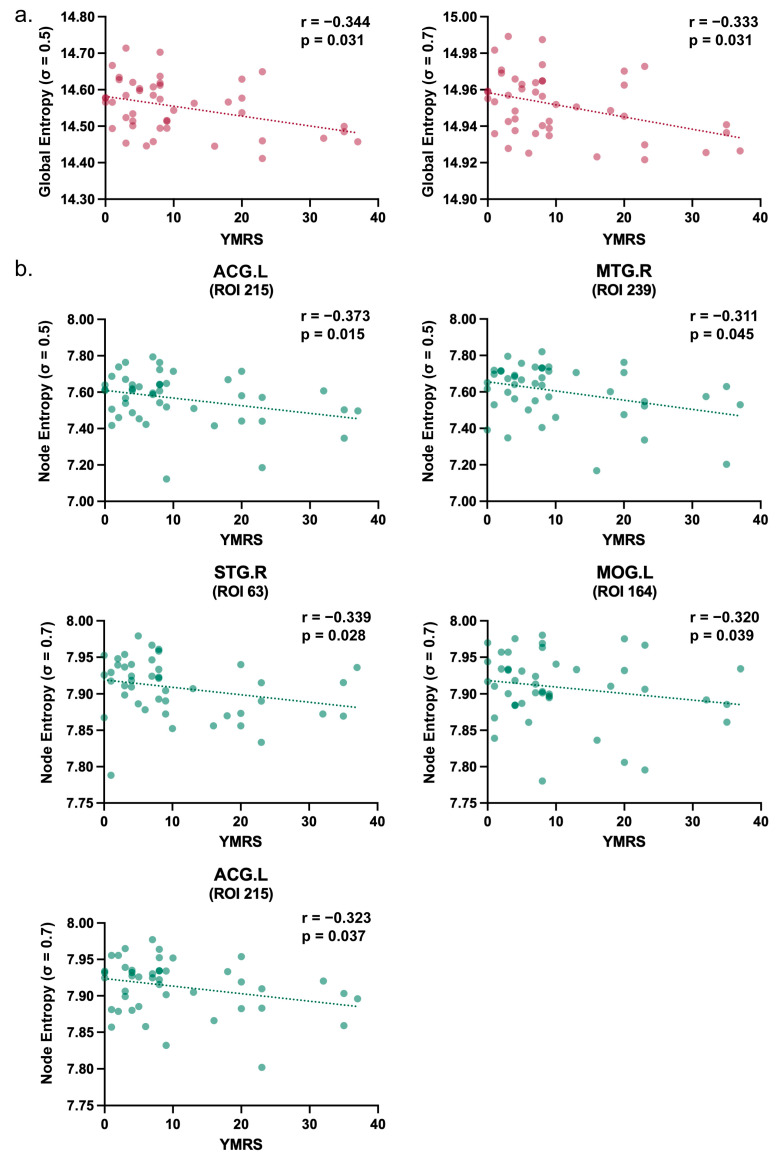
Correlations between entropy and manic symptoms (YMRS). Plots display exploratory partial correlations controlling for age, sex, and mean FD (*p* < 0.05, uncorrected). (**a**) Global entropy and (**b**) nodal entropy both showed significant negative correlations with YMRS scores. No significant correlations were found for HAMD.

At the global level (Figure 5a), significant negative correlations were identified between global entropy and YMRS scores at *σ* = 0.5 (*r* = −0.344, *p* = 0.031) and *σ* = 0.7 (*r* = −0.333, *p* = 0.031). These findings indicate that increased manic severity is associated with a relative reduction in global network entropy. In contrast, no significant correlation was observed at *σ* = 0.3. This parameter-dependent pattern mirrors the group-level findings, suggesting that the distributional shifts linked to manic symptoms are most discernible at intermediate and broader kernel scales.

At the nodal level (Figure 5b), consistent with the global findings, significant negative correlations were identified between nodal entropy and YMRS scores in specific brain regions. These associations were most prominent at *σ* = 0.5 and *σ* = 0.7. Specifically, at *σ* = 0.5, higher manic symptom severity was associated with reduced entropy in the ACG.L (*r* = −0.373, *p* = 0.015) and MTG.R (*r* = −0.311, *p* = 0.045). Similarly, at *σ* = 0.7, negative correlations were identified in the STG.R (*r* = −0.339, *p* = 0.028), MOG.L (*r* = −0.320, *p* = 0.039) and ACG.L (*r* = −0.323, *p* = 0.037). Regarding depressive symptoms, no significant correlations were observed between nodal entropy and HAMD scores in any of the identified regions.

These findings indicate that while patients with BD generally exhibit higher entropy (dedifferentiation) than controls, increased manic severity within the patient group is associated with a relative reduction in entropy, potentially reflecting a pathological “locking-in” of specific circuits.

## 4. Discussion

This study applied a kernel-transformed functional connectivity (FC) entropy framework to characterize multiscale brain network dysregulation in BD. By performing a hierarchical analysis at the global, modular, and nodal levels—and employing a rigorous statistical approach that controlled for head motion (mean FD), age, and sex via ANCOVA—we identified widespread network dedifferentiation that is closely linked to clinical phenotypes. Importantly, this strict control strategy ensures that the observed alterations are driven by underlying neurobiological mechanisms rather than confounding artifacts [35,36].

### 4.1. Advancement of Kernel-Transformed FC Entropy

Traditional functional connectivity analysis typically relies on Pearson correlation matrices, which, while effective for quantifying coupling strength, are often limited by their dense and noisy nature [37]. As previously established, the contrast between strong and weak connections in raw Pearson matrices is frequently insufficient to reveal subtle distributional shifts, potentially obscuring critical topological properties such as the balance between integration and segregation [38,39]. To address this limitation, our framework employs a kernel-transformed similarity mapping. Conceptually, this transformation acts as a reweighting filter designed to expand the contrast of the connectivity profile [40]. By enhancing the separation between high-affinity (strong) and low-affinity (weak) connections, the kernelization effectively suppresses background noise while highlighting topologically significant links [41]. This contrast expansion is critical because it transforms a noisy, dense matrix into a clearer affinity structure mapping, thereby rendering the subsequent Shannon entropy metric significantly more sensitive to detecting pathological alterations in network uniformity and dedifferentiation.

The efficacy of this contrast enhancement mechanism is strictly governed by the kernel width. We selected *σ* = 0.5 as the primary setting because empirical testing indicated it provides the optimal balance for this reweighting process. A smaller width (e.g., *σ* = 0.3) tends to act as an overly aggressive threshold, disproportionately penalizing moderate connections and losing topologically relevant weak links [42]. Conversely, a larger width (e.g., *σ* = 0.7) yields insufficient contrast expansion, failing to suppress noise effectively and resulting in an overly smoothed distribution. Therefore, *σ* = 0.5 serves as an ideal soft threshold parameter, preserving the richness of the weight distribution while effectively attenuating noise to reveal the intrinsic network organization.

### 4.2. Multiscale Network Dysregulation in Bipolar Disorder

Our findings consistently demonstrate significantly higher entropy in patients with BD across multiple scales, supporting the “network dedifferentiation” hypothesis. In the context of weighted brain networks, Shannon entropy quantifies the uniformity of the weight distribution [43]. A healthy, efficiently organized brain network typically maintains a highly non-uniform distribution, reflecting a vital balance between strong intramodular segregation and specific intermodular integration [44]. The elevated entropy observed in BD indicates a flattened connectivity profile, suggesting that the boundaries between functional specializations have become blurred [45]. To further clarify the physical significance of elevated entropy, we employed the Gini coefficient as a complementary descriptor of the connectivity weight distribution. Our analysis (illustrated in Supplementary Figure S1) revealed a significantly lower Gini coefficient in the BD group, confirming a more uniform or flattened connectivity profile. This shift underscores a state of network dedifferentiation, where the topological distinction between strong functional hubs and weak background connections is diminished.

At the global level, the significant increase in entropy (particularly at *σ* = 0.5 and 0.7) reflects a systemic loss of network specificity. This whole-brain dedifferentiation suggests that the brain network in BD has shifted toward a more random or uniform configuration, potentially increasing the metabolic or computational cost required to maintain stable cognitive states [46]. Importantly, these global differences remained significant after strictly controlling for mean FD in the ANCOVA models, providing robust evidence that the results are driven by intrinsic neurobiology rather than motion artifacts [47].

Descending to the modular level, we observed prominent entropy increases specifically within the Default Mode Network (DMN), Ventral Attention Network (VAN), and Dorsal Attention Network (DAN). These networks are critical for self-referential processing and top-down attentional control [48]. The elevated entropy within these modules indicates that their internal connectivity is less segregated in patients with BD, suggesting a loss of functional precision that may render these core systems more prone to cross-network interference and noise [49].

Finally, at the nodal level, the topological alterations were characterized by a widespread distribution rather than being confined to a single focal region. As detailed in our results, regions exhibiting significantly higher entropy extended beyond high-order cognitive networks to include the Somatosensory-Motor Network (MSN) and the Salience Network (SAL). The involvement of these diverse systems is noteworthy: elevated entropy in the MSN implies instability even in fundamental sensory processing, while alterations in the SAL suggest impaired filtering of relevant internal and external stimuli [50]. This pervasive nodal distribution aligns with the global findings, providing granular evidence that dedifferentiation in BD acts as a broad-spectrum dysregulation. Such widespread alterations compromise the functional segregation required for processes ranging from basic sensorimotor integration to complex executive control.

### 4.3. Clinical Implications of Kernel-Transformed FC Entropy

To investigate the clinical utility of our metrics, we conducted exploratory partial correlation analyses to assess the associations between entropy measures and symptom severity, stringently controlling for potential confounders including age, sex, and mean FD.

Regarding manic symptoms, global entropy and specific nodal entropy (e.g., in the ACG.R and MTG.R) exhibited significant negative correlations with YMRS scores. This reveals a nuanced biological paradox: while patients with BD generally exhibit higher network uncertainty than controls, an increase in manic severity within the patient group is associated with a relative reduction in entropy. We propose that this reflects a pathological “locking-in” of specific neural circuits. Clinically, this aligns with the cognitive and behavioral rigidity often observed in mania, where connectivity weights become excessively concentrated along restricted, stereotyped paths. This reduces the overall uniformity of the distribution (lower entropy) but occurs at the cost of the functional flexibility required for adaptive behavior [51]. It is worth noting that these significant clinical correlations were observed at *σ* = 0.5 and *σ* = 0.7, whereas no significant association was detected at *σ* = 0.3. This parameter-dependent pattern mirrors the group-level differences, suggesting that the sparse connectivity structure induced by a small kernel width may be insufficient to capture the subtle topological shifts linked to symptom severity.

In contrast to the manic domain, no significant correlations were observed between nodal entropy and depressive symptom severity (HAMD scores) in the identified regions. This dissociation suggests that distinct mood dimensions in BD may rest upon different underlying neural mechanisms [52]. While mania appears linked to a pathological over-structuring of connectivity (reduced entropy), depressive symptomatology might be driven by neurochemical or metabolic factors that do not manifest as perceptible alterations in the uniformity of connection weights. Alternatively, the network correlates of depression may be more subtle or localized within subcortical circuits not fully captured by the current nodal analysis. These findings indicate that kernel-transformed FC entropy may be particularly sensitive to the hyper-connective and rigid states characteristic of mania, rather than the hypo-activity often associated with depression.

### 4.4. Limitations

Several limitations should be considered when interpreting these results. First, regarding preprocessing choices, we applied Global Signal Regression (GSR) to mitigate widespread physiological noise and strictly controlled for head motion. While GSR effectively attenuates global artifacts and enhances the topological contrast required for entropy analysis, it is known to mathematically shift the distribution of correlation coefficients toward zero. Therefore, the entropy values reported here should be interpreted specifically within the context of GSR-corrected functional connectivity, reflecting the relative heterogeneity of the network weights rather than global synchronization levels.

Second, regarding medication effects, the potential confounding influence of psychotropic medications cannot be fully excluded. Due to the lack of detailed quantitative data on medication dosages and the heterogeneity of treatment regimens within our sample, we were unable to perform fine-grained analyses to separate specific drug effects from underlying disease pathology. Consequently, it remains unclear whether the observed entropy alterations are purely intrinsic to the illness or partially modulated by pharmacological interventions. Future studies involving treatment-naive cohorts are warranted to clarify this issue.

Third, our parameter selection was constrained to a discrete set of kernel widths. Sensitivity analyses revealed that the identified network dedifferentiation is a scale-dependent phenomenon. Robust group differences in global, nodal, and modular entropy were consistently observed at intermediate and coarse scales (*σ* ≥ 0.5). However, these effects were substantially attenuated at the finest scale (*σ* = 0.3), where no significant global difference was detected, and sparse effects were limited to only 3 nodes and 1 module. This suggests that the topological reorganization in BD is best characterized at scales that filter fine-grained local fluctuations. We explicitly acknowledge that nodal-level findings are parameter-dependent, as their statistical significance relies on selecting an optimal bandwidth. Future studies could employ data-driven optimization strategies to further refine scale selection for specific sub-networks.

Finally, a specific methodological constraint of our modular analysis was the exclusive focus on intramodular connections. This design choice was deliberately made to isolate and quantify the internal stability and segregation of functional communities. Methodologically, including intermodular edges in the entropy calculation would confound two distinct topological properties: network segregation and network integration. By focusing strictly on intramodular weights, we ensured an unambiguous interpretation of modular dedifferentiation, albeit at the cost of overlooking potential alterations in inter-regional communication between disparate functional systems [53]. Future work could extend this framework to calculate inter-modular entropy to specifically address this gap.

## 5. Conclusions

In conclusion, this study demonstrates that kernel-transformed functional connectivity entropy provides a flexible and robust method for quantifying brain network dedifferentiation in BD. By employing a contrast-enhancing reweighting mechanism, this approach overcomes the limitations of dense linear correlation matrices, enabling the detection of subtle distributional shifts that characterize network dedifferentiation. Our multiscale analyses revealed widespread entropy increases in patients with BD, particularly within core cognitive and attentional systems (DMN, VAN, and DAN), signaling a loss of functional segregation. Furthermore, the identification of a negative correlation between entropy and manic severity offers novel insights into the “ locking-in” dynamics of mania, effectively distinguishing it from the general network dysregulation. Collectively, these findings support the potential utility of kernel-transformed entropy as a candidate neuroimaging metric for characterizing BD-related dysregulation, offering a methodological foundation for future research into the state-dependent topology of psychiatric disorders.

## Figures and Tables

**Table 1:** Demographic and clinical characteristics ^a^.

Characteristic	Group (BD/NCs = 45/45)		*p*-Value
	Patients with BD	Normal Controls	
Age (years)	35.53 ± 9.21 (21–50)	35.56 ± 9.18 (21–50)	*p* = 0.991 ^b^
Male/Female	26/19	26/19	*p* = 1.000 ^c^
Mean FD (mm)	0.11 ± 0.06 (0.03–0.25)	0.13 ± 0.08 (0.03–0.36)	*p* = 0.191 ^d^
YMRS ^e^	10.49 ± 11.22 (0–37)	—	—
HAMD ^f^	11.62 ± 8.43 (0–32)	—	—

BD: Bipolar Disorder; NC: Normal Controls; YMRS: Young Mania Rating Scale; HAMD: 17-item Hamilton Depression Rating Scale. ^a^ Unless otherwise indicated, data are expressed as mean ± SD (minimum–maximum). ^b^ The *p*-value was obtained using an independent sample *t*-test. ^c^ The *p*-value was obtained using a Pearson χ^2^ test. ^d^ The *p*-value was obtained using an independent sample *t*-test. ^e^ YMRS scores were available only for patients with BD. ^f^ HAMD scores were available only for patients with BD.

**Table 2:** Statistical information for ROIs showing significant differences between NCs and BD patients when σ=0.5.

No.	ROI	Module	Peak MNI	*F*-Value	*p*-Value	*q*-Value	*η_p_* ^2^
20	SMG.L	MSN	(−54, −23, 43)	12.730	0.001	0.028	0.130
21	PreCG.R	MSN	(29, −17, 71)	11.734	0.001	0.029	0.121
24	PoCG.L	MSN	(−40, −19, 54)	10.558	0.002	0.031	0.110
29	PreCG.R	MSN	(44, −8, 57)	11.141	0.001	0.030	0.116
33	PoCG.L	MSN	(−45, −32, 47)	9.790	0.002	0.032	0.103
36	PoCG.R	MSN	(42, −20, 55)	16.787	<0.001	0.012	0.165
37	PreCG.L	MSN	(−38, −15, 69)	8.283	0.005	0.045	0.089
41	PreCG.R	MSN	(38, −17, 45)	9.458	0.003	0.032	0.100
63	STG.R	AUD	(58, −16, 7)	9.123	0.003	0.033	0.097
79	MTG.L	DMN	(−46, −61, 21)	10.797	0.001	0.031	0.113
80	MOG.R	DMN	(43, −72, 28)	9.624	0.003	0.032	0.102
86	ANG.L	DMN	(−44, −65, 35)	10.543	0.002	0.031	0.110
88	PCUN.L	DMN	(−7, −55, 27)	9.742	0.002	0.032	0.103
94	DCG.L	DMN	(−2, −37, 44)	10.078	0.002	0.032	0.106
95	PCUN.R	DMN	(11, −54, 17)	9.345	0.003	0.033	0.099
96	ANG.R	DMN	(52, −59, 36)	9.237	0.003	0.033	0.098
112	ORBmid.L	DMN	(−2, 38, 36)	8.641	0.004	0.039	0.092
113	ACG.L	DMN	(−3, 42, 16)	15.389	0.000	0.012	0.153
122	ACG.R	DMN	(12, 36, 20)	10.460	0.002	0.031	0.110
130	ANG.R	DMN	(47, −50, 29)	15.697	0.000	0.012	0.156
164	MOG.L	VIS	(−42, −74, 0)	9.683	0.003	0.032	0.102
177	IPL.L	FPN	(−53, −49, 43)	11.432	0.001	0.029	0.119
195	ANG.L	FPN	(−42, −55, 45)	11.851	0.001	0.029	0.122
208	INS.L	SAL	(−35, 20, 0)	19.712	<0.001	0.007	0.188
215	ACG.L	SAL	(0, 30, 27)	10.245	0.002	0.032	0.108
221	MFG.R	SAL	(2, −24, 30)	9.108	0.003	0.033	0.09
239	MTG.R	VAN	(51, −29, −4)	8.064	0.006	0.048	0.087
259	IPL.L	DAN	(−33, −46, 47)	12.613	0.001	0.028	0.129
264	PreCG.R	DAN	(29, −5, 54)	11.454	0.001	0.029	0.119

Note: Some anatomical labels (e.g., PreCG.R) appear multiple times; these represent distinct ROIs with different MNI coordinates within the Power-264 atlas.

## Data Availability

The data used in this study were obtained from the University of California, Los Angeles (UCLA) Consortium for Neuropsychiatric Phenomics LA5c open access dataset. This data is available in OpenNeuro at doi: 10.18112/openneuro.ds000030.v1.0.0, reference number: ds000030.

## Supplementary Materials

The following supporting information can be downloaded at https://www.mdpi.com/article/10.3390/brainsci16020208/s1, Figure S1. Comparison of Gini coefficients between BD and NC groups. This complementary metric validates the network dedifferentiation characterized by increased Shannon entropy. (* *p* < 0.05, *** *p* < 0.001). Table S1. Node entropy group differences at *σ* = 0.3 (BD vs. NCs). Regions showing statistically significant differences (FDR corrected *q* < 0.05) are marked in bold and red. Table S2. Node entropy group differences at *σ* = 0.5 (BD vs. NCs). Regions showing statistically significant differences (FDR corrected *q* < 0.05) are marked in bold and red. Table S3. Node entropy group differences at *σ* = 0.7 (BD vs. NCs). Regions showing statistically significant differences (FDR corrected *q* < 0.05) are marked in bold and red.
