# Supplementary material for: Kernel-Transformed Functional Connectivity Entropy Reveals Network Dedifferentiation in Bipolar Disorder
Source: Brain Sci. 2026 Feb 10;16(2):208. doi: 10.3390/brainsci16020208 (PMC12938087; doi:10.3390/brainsci16020208)
Supplement: Supplementary file 1 [file brainsci-16-00208-s001.zip › Supplementary_Figure S1.pdf]

Supplementary Materials

Note 1: Relationship between Entropy and Gini Coefficient

To strictly interpret the physical meaning of the elevated functional connectivity entropy, we employed the Gini coefficient, a standard measure of statistical dispersion used to represent the inequality of a distribution. In the context of weighted networks, Shannon entropy and the Gini coefficient generally exhibit an inverse relationship. A system with maximum entropy corresponds to a uniform distribution, yielding a Gini coefficient of zero (perfect equality). Conversely, a segregated, highly non-uniform network—typical of healthy brain organization—exhibits low entropy and a high Gini coefficient (high inequality).

To empirically validate this theoretical relationship, we calculated the Gini coefficients of the connectivity weights for both groups and visualized the distributions using box plots (Figure S1). As illustrated, the BD group exhibited significantly lower Gini coefficients compared to NCs across optimal kernel scales. This empirical finding aligns with the entropy results and reflects a flattening of the connectivity profile, where the topological distinction between strong, hub-like connections and weak background associations is diminished. This relationship provides robust quantitative support for the network dedifferentiation hypothesis.

We performed independent samples t-tests between BD and NC groups. The analysis confirmed a significant reduction in the Gini coefficient for the BD group across all tested kernel widths: $\sigma = 0.3$: $t = 2.267$, $p = 0.026$; $\sigma = 0.5$: $t = 3.676$, $p < 0.001$; $\sigma = 0.7$: $t = 3.748$, $p < 0.001$. These results statistically validate the "flattening" of the connectivity weight distribution in BD patients.

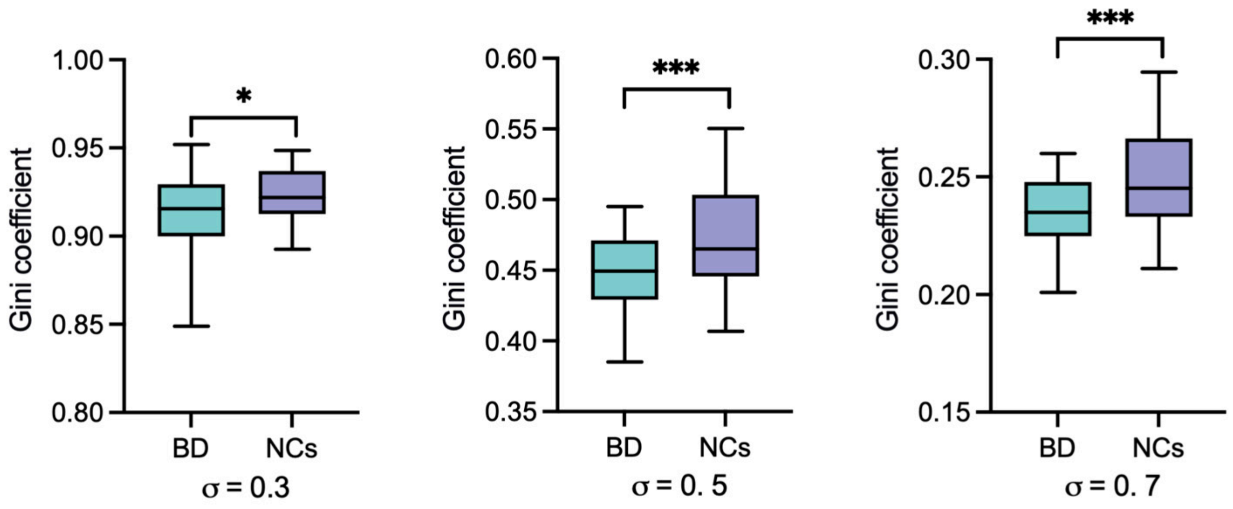

Figure S1. Comparison of Gini coefficients between BD and NC groups. This complementary metric validates the network dedifferentiation characterized by increased Shannon entropy. (*$p <$ 0.05, *** $p < 0.001$).
