# Supplementary material for: Kernel-Transformed Functional Connectivity Entropy Reveals Network Dedifferentiation in Bipolar Disorder
Source: Brain Sci. 2026 Feb 10;16(2):208. doi: 10.3390/brainsci16020208 (PMC12938087; doi:10.3390/brainsci16020208)
Supplement: Supplementary file 1 [file brainsci-16-00208-s001.zip › Supplementary_Tables S1-S3.pdf]

Supplementary Materials

Table S1. Node entropy group differences at σ = 0.3 (BD vs. NCs). Regions showing statistically significant differences (FDR corrected $q < 0.05$) are marked in bold and red.

No.	ROI	Module	Peak MNI	F-value	p-value	q-value	η_p^2
1	No	No	(-25, -98, -12)	2.849	0.095	0.386	0.032
2	No	No	(27, -97, -13)	0.067	0.796	0.891	0.001
3	No	No	(24, 32, -18)	0.517	0.474	0.745	0.006
4	No	No	(-56, -45, -24)	0.855	0.358	0.642	0.010
5	No	No	(8, 41, -24)	1.163	0.284	0.604	0.013
6	No	No	(-21, -22, -20)	0.458	0.500	0.753	0.005
7	No	No	(17, -28, -17)	0.004	0.951	0.977	<0.001
8	No	No	(-37, -29, -26)	2.119	0.149	0.480	0.024
9	No	No	(65, -24, -19)	1.665	0.200	0.517	0.019
10	No	No	(52, -34, -27)	0.078	0.780	0.887	0.001
11	No	No	(55, -31, -17)	<0.001	0.991	0.995	<0.001
12	No	No	(34, 38, -12)	3.252	0.075	0.324	0.037
13	PCUN.L	MSN	(-7, -52, 61)	2.341	0.130	0.450	0.027
14	DCG.L	MSN	(-14, -18, 40)	0.113	0.738	0.882	0.001
15	DCG.L	MSN	(0, -15, 47)	3.814	0.054	0.301	0.043
16	DCG.R	MSN	(10, -2, 45)	3.915	0.051	0.301	0.044
17	PCL.L	MSN	(-7, -21, 65)	1.997	0.161	0.480	0.023
18	PCL.L	MSN	(-7, -33, 72)	0.080	0.778	0.887	0.001
19	PoCG.R	MSN	(13, -33, 75)	1.576	0.213	0.520	0.018
20	SMG.L	MSN	(-54, -23, 43)	8.207	0.005	0.139	0.088
21	PreCG.R	MSN	(29, -17, 71)	2.031	0.158	0.480	0.023
22	PCUN.R	MSN	(10, -46, 73)	0.235	0.629	0.826	0.003
23	PoCG.L	MSN	(-23, -30, 72)	2.144	0.147	0.480	0.025
24	PoCG.L	MSN	(-40, -19, 54)	6.246	0.014	0.211	0.068
25	PoCG.R	MSN	(29, -39, 59)	1.142	0.288	0.609	0.013
26	PoCG.R	MSN	(50, -20, 42)	1.877	0.174	0.485	0.022
27	PoCG.L	MSN	(-38, -27, 69)	3.935	0.051	0.301	0.044
28	PreCG.R	MSN	(20, -29, 60)	3.337	0.071	0.320	0.038
29	PreCG.R	MSN	(44, -8, 57)	9.728	0.002	0.093	0.103
30	PoCG.L	MSN	(-29, -43, 61)	1.745	0.190	0.504	0.020
31	SMA.R	MSN	(10, -17, 74)	0.141	0.708	0.865	0.002
32	PoCG.R	MSN	(22, -42, 69)	0.325	0.570	0.805	0.004
33	PoCG.L	MSN	(-45, -32, 47)	2.144	0.147	0.480	0.025
34	PoCG.L	MSN	(-21, -31, 61)	7.016	0.010	0.211	0.076
35	PCL.L	MSN	(-13, -17, 75)	0.521	0.472	0.745	0.006
36	PoCG.R	MSN	(42, -20, 55)	16.250	<0.001	0.016	0.160

37	PreCG.L	MSN	(-38, -15, 69)	10.108	0.002	0.091	0.106
38	SPG.L	MSN	(-16, -46, 73)	0.763	0.385	0.664	0.009
39	PCL.R	MSN	(2, -28, 60)	6.653	0.012	0.211	0.073
40	SMA.R	MSN	(3, -17, 58)	8.739	0.004	0.118	0.093
**41**	**PreCG.R**	**MSN**	**(38, -17, 45)**	**17.072**	**<0.001**	**0.016**	**0.167**
42	No	No	(-49, -11, 35)	4.671	0.033	0.272	0.052
43	No	No	(36, -9, 14)	0.096	0.758	0.887	0.001
44	No	No	(51, -6, 32)	4.799	0.031	0.268	0.053
45	No	No	(-53, -10, 24)	2.133	0.148	0.480	0.024
46	No	No	(66, -8, 25)	1.501	0.224	0.520	0.017
47	SMA.L	CON	(-3, 2, 53)	1.010	0.318	0.612	0.012
48	SMG.R	CON	(54, -28, 34)	0.982	0.325	0.612	0.011
49	SFGdor.R	CON	(19, -8, 64)	1.423	0.236	0.529	0.016
50	SFGdor.L	CON	(-16, -5, 71)	0.693	0.408	0.683	0.008
51	DCG.L	CON	(-10, -2, 42)	1.078	0.302	0.612	0.013
52	INS.R	CON	(37, 1, -4)	2.742	0.101	0.406	0.031
53	SMA.R	CON	(13, -1, 70)	0.071	0.791	0.889	0.001
54	SMA.R	CON	(7, 8, 51)	3.299	0.073	0.320	0.037
55	ROL.L	CON	(-45, 0, 9)	0.077	0.782	0.887	0.001
56	INS.R	CON	(49, 8, -1)	0.866	0.355	0.641	0.010
57	PUT.L	CON	(-34, 3, 4)	4.251	0.042	0.294	0.048
58	TPOsup.L	CON	(-51, 8, -2)	0.488	0.487	0.747	0.006
59	DCG.L	CON	(-5, 18, 34)	0.426	0.516	0.765	0.005
60	INS.R	CON	(36, 10, 1)	0.395	0.531	0.776	0.005
61	Heschl_R	AUD	(32, -26, 13)	3.087	0.083	0.351	0.035
62	STG.R	AUD	(65, -33, 20)	0.288	0.593	0.817	0.003
63	STG.R	AUD	(58, -16, 7)	1.661	0.201	0.517	0.019
64	ROL.L	AUD	(-38, -33, 17)	0.466	0.497	0.753	0.005
65	STG.L	AUD	(-60, -25, 14)	0.012	0.912	0.955	<0.001
66	STG.L	AUD	(-49, -26, 5)	3.361	0.070	0.320	0.038
67	ROL.R	AUD	(43, -23, 20)	3.364	0.070	0.320	0.038
68	SMG.L	AUD	(-50, -34, 26)	4.530	0.036	0.273	0.051
69	SMG.L	AUD	(-53, -22, 23)	3.792	0.055	0.301	0.043
70	HES.L	AUD	(-55, -9, 12)	1.876	0.174	0.485	0.022
71	ROL.R	AUD	(56, -5, 13)	0.629	0.430	0.714	0.007
72	SMG.R	AUD	(59, -17, 29)	0.017	0.897	0.955	<0.001
73	ROL.L	AUD	(-30, -27, 12)	0.091	0.763	0.887	0.001
74	MOG.L	DMN	(-41, -75, 26)	1.774	0.186	0.504	0.020
75	ORBmid.R	DMN	(6, 67, -4)	1.496	0.225	0.520	0.017
76	Rectus_R	DMN	(8, 48, -15)	1.611	0.208	0.520	0.019
77	LING.L	DMN	(-13, -40, 1)	0.230	0.633	0.826	0.003
78	ORBsup.L	DMN	(-18, 63, -9)	3.855	0.053	0.301	0.043
79	MTG.L	DMN	(-46, -61, 21)	6.242	0.014	0.211	0.068

80	MOG.R	DMN	(43, -72, 28)	2.870	0.094	0.386	0.033
81	TPOmid.L	DMN	(-44, 12, -34)	3.802	0.054	0.301	0.043
82	TPOmid.R	DMN	(46, 16, -30)	1.924	0.169	0.480	0.022
83	ITG.L	DMN	(-68, -23, -16)	0.910	0.343	0.633	0.011
84	No	No	(-58, -26, -15)	2.505	0.117	0.436	0.029
85	No	No	(27, 16, -17)	0.361	0.550	0.789	0.004
86	ANG.L	DMN	(-44, -65, 35)	2.057	0.155	0.480	0.024
87	IPL.L	DMN	(-39, -75, 44)	0.892	0.348	0.637	0.010
88	PCUN.L	DMN	(-7, -55, 27)	5.505	0.021	0.246	0.061
89	PCUN.R	DMN	(6, -59, 35)	0.156	0.694	0.860	0.002
90	PCUN.L	DMN	(-11, -56, 16)	6.542	0.012	0.211	0.071
91	PCUN.L	DMN	(-3, -49, 13)	2.028	0.158	0.480	0.023
92	PCG.R	DMN	(8, -48, 31)	0.429	0.514	0.765	0.005
93	PCUN.R	DMN	(15, -63, 26)	2.698	0.104	0.410	0.031
94	DCG.L	DMN	(-2, -37, 44)	1.508	0.223	0.520	0.017
95	PCUN.R	DMN	(11, -54, 17)	11.933	0.001	0.053	0.123
96	ANG.R	DMN	(52, -59, 36)	0.393	0.532	0.776	0.005
97	SFGdor.R	DMN	(23, 33, 48)	1.501	0.224	0.520	0.017
98	ORBmid.L	DMN	(-10, 39, 52)	0.353	0.554	0.791	0.004
99	SFGdor.L	DMN	(-16, 29, 53)	1.472	0.228	0.524	0.017
100	MFG.L	DMN	(-35, 20, 51)	0.994	0.322	0.612	0.012
101	SFGdor.R	DMN	(22, 39, 39)	0.044	0.834	0.918	0.001
102	SFGdor.R	DMN	(13, 55, 38)	2.419	0.124	0.450	0.028
103	SFGdor.L	DMN	(-10, 55, 39)	0.698	0.406	0.683	0.008
104	SFGdor.L	DMN	(-20, 45, 39)	0.297	0.587	0.816	0.003
105	SFGmed.R	DMN	(6, 54, 16)	5.414	0.022	0.246	0.060
106	SFGmed.R	DMN	(6, 64, 22)	0.249	0.619	0.817	0.003
107	ACG.L	DMN	(-7, 51, -1)	5.058	0.027	0.268	0.056
108	SFGmed.R	DMN	(9, 54, 3)	0.080	0.778	0.887	0.001
109	ORBmid.L	DMN	(-3, 44, -9)	0.278	0.599	0.817	0.003
110	ORBmid.R	DMN	(8, 42, -5)	1.565	0.214	0.520	0.018
111	ACG.L	DMN	(-11, 45, 8)	0.563	0.455	0.736	0.007
112	ORBmid.L	DMN	(-2, 38, 36)	1.225	0.272	0.583	0.014
113	ACG.L	DMN	(-3, 42, 16)	11.623	0.001	0.053	0.120
114	SFGdor.L	DMN	(-20, 64, 19)	0.774	0.382	0.664	0.009
115	ORBmid.L	DMN	(-8, 48, 23)	0.083	0.774	0.887	0.001
116	MTG.R	DMN	(65, -12, -19)	1.791	0.184	0.504	0.021
117	MTG.L	DMN	(-56, -13, -10)	3.581	0.062	0.313	0.040
118	MTG.L	DMN	(-58, -30, -4)	3.442	0.067	0.320	0.039
119	MTG.R	DMN	(65, -31, -9)	0.002	0.965	0.984	<0.001
120	MTG.L	DMN	(-68, -41, -5)	0.008	0.930	0.969	<0.001
121	SFGdor.R	DMN	(13, 30, 59)	0.013	0.910	0.955	<0.001
122	ACG.R	DMN	(12, 36, 20)	6.276	0.014	0.211	0.069

123	MTG.R	DMN	(52, -2, -16)	2.323	0.131	0.450	0.027
124	PHG.L	DMN	(-26, -40, -8)	4.787	0.031	0.268	0.053
125	FFG.R	DMN	(27, -37, -13)	3.673	0.059	0.304	0.041
126	FFG.L	DMN	(-34, -38, -16)	1.609	0.208	0.520	0.019
127	Cerebelum	DMN	(28, -77, -32)	4.449	0.038	0.278	0.050
128	TPOmid.R	DMN	(52, 7, -30)	5.451	0.022	0.246	0.060
129	MTG.L	DMN	(-53, 3, -27)	2.396	0.125	0.450	0.027
130	ANG.R	DMN	(47, -50, 29)	3.712	0.057	0.303	0.042
131	MTG.L	DMN	(-49, -42, 1)	<0.001	0.983	0.991	<0.001
132	No	No	(-31, 19, -19)	0.094	0.760	0.887	0.001
133	No	No	(-2, -35, 31)	0.570	0.452	0.736	0.007
134	No	No	(-7, -71, 42)	0.497	0.483	0.745	0.006
135	No	No	(11, -66, 42)	0.381	0.539	0.780	0.004
136	No	No	(4, -48, 51)	1.328	0.252	0.560	0.015
137	ORBinf.L	DMN	(-46, 31, -13)	2.358	0.128	0.450	0.027
138	SMA.L	VAN	(-10, 11, 67)	1.524	0.220	0.520	0.018
139	ORBinf.R	DMN	(49, 35, -12)	3.031	0.085	0.357	0.034
140	No	No	(8, -91, -7)	0.617	0.434	0.717	0.007
141	No	No	(17, -91, -14)	0.031	0.861	0.938	<0.001
142	No	No	(-12, -95, -13)	1.740	0.191	0.504	0.020
143	LING.R	VIS	(18, -47, -10)	2.542	0.115	0.432	0.029
144	MOG.R	VIS	(40, -72, 14)	3.923	0.051	0.301	0.044
145	CAL.R	VIS	(8, -72, 11)	4.094	0.046	0.297	0.046
146	CAL.L	VIS	(-8, -81, 7)	0.959	0.330	0.619	0.011
147	MOG.L	VIS	(-28, -79, 19)	0.994	0.322	0.612	0.012
148	LING.R	VIS	(20, -66, 2)	0.017	0.896	0.955	<0.001
149	MOG.L	VIS	(-24, -91, 19)	0.504	0.480	0.745	0.006
150	FFG.R	VIS	(27, -59, -9)	4.135	0.045	0.297	0.046
151	LING.L	VIS	(-15, -72, -8)	0.166	0.685	0.860	0.002
152	CAL.L	VIS	(-18, -68, 5)	0.526	0.470	0.745	0.006
153	IOG.R	VIS	(43, -78, -12)	1.956	0.166	0.480	0.022
154	IOG.L	VIS	(-47, -76, -10)	1.746	0.190	0.504	0.020
155	SOG.L	VIS	(-14, -91, 31)	0.005	0.945	0.974	<0.001
156	SOG.R	VIS	(15, -87, 37)	0.781	0.379	0.664	0.009
157	MOG.R	VIS	(29, -77, 25)	0.056	0.813	0.906	0.001
158	LING.R	VIS	(20, -86, -2)	0.986	0.323	0.612	0.011
159	CUN.R	VIS	(15, -77, 31)	0.162	0.688	0.860	0.002
160	LING.L	VIS	(-16, -52, -1)	0.454	0.502	0.753	0.005
161	ITG.R	VIS	(42, -66, -8)	0.079	0.780	0.887	0.001
162	SOG.R	VIS	(24, -87, 24)	0.688	0.409	0.683	0.008
163	CUN.R	VIS	(6, -72, 24)	4.646	0.034	0.272	0.052
164	MOG.L	VIS	(-42, -74, 0)	5.917	0.017	0.229	0.065
165	Cerebelum	VIS	(26, -79, -16)	0.005	0.941	0.974	<0.001

166	CUN.L	VIS	(-16, -77, 34)	0.377	0.541	0.780	0.004
167	CUN.L	VIS	(-3, -81, 21)	0.023	0.881	0.953	<0.001
168	MOG.L	VIS	(-40, -88, -6)	0.128	0.721	0.873	0.002
169	MOG.R	VIS	(37, -84, 13)	0.201	0.655	0.843	0.002
170	CAL.R	VIS	(6, -81, 6)	0.553	0.459	0.736	0.006
171	MOG.L	VIS	(-26, -90, 3)	2.541	0.115	0.432	0.029
172	FFG.L	VIS	(-33, -79, -13)	1.297	0.258	0.568	0.015
173	MOG.R	VIS	(37, -81, 1)	0.998	0.321	0.612	0.012
174	PreCG.L	FPN	(-44, 2, 46)	0.315	0.576	0.809	0.004
175	IFGtriang.R	FPN	(48, 25, 27)	1.061	0.306	0.612	0.012
176	ORBinf.L	FPN	(-47, 11, 23)	<0.001	0.997	0.997	<0.001
177	IPL.L	FPN	(-53, -49, 43)	1.562	0.215	0.520	0.018
178	MFG.L	FPN	(-23, 11, 64)	0.074	0.786	0.887	0.001
179	ITG.R	FPN	(58, -53, -14)	1.054	0.307	0.612	0.012
180	ORBsup.R	FPN	(24, 45, -15)	0.828	0.365	0.652	0.010
181	ORBmid.R	FPN	(34, 54, -13)	0.347	0.557	0.791	0.004
182	No	No	(-21, 41, -20)	0.500	0.481	0.745	0.006
183	No	No	(-18, -76, -24)	0.038	0.845	0.926	<0.001
184	No	No	(17, -80, -34)	1.105	0.296	0.612	0.013
185	No	No	(35, -67, -34)	0.187	0.666	0.847	0.002
186	PreCG.R	FPN	(47, 10, 33)	4.928	0.029	0.268	0.055
187	PreCG.L	FPN	(-41, 6, 33)	0.075	0.785	0.887	0.001
188	MFG.L	FPN	(-42, 38, 21)	0.260	0.611	0.817	0.003
189	MFG.R	FPN	(38, 43, 15)	0.160	0.691	0.860	0.002
190	SMG.R	FPN	(49, -42, 45)	0.394	0.532	0.776	0.005
191	SPG.L	FPN	(-28, -58, 48)	0.119	0.731	0.878	0.001
192	IPL.R	FPN	(44, -53, 47)	0.251	0.618	0.817	0.003
193	MFG.R	FPN	(32, 14, 56)	0.869	0.354	0.641	0.010
194	ANG.R	FPN	(37, -65, 40)	0.052	0.821	0.910	0.001
195	ANG.L	FPN	(-42, -55, 45)	0.910	0.343	0.633	0.011
196	MFG.R	FPN	(40, 18, 40)	0.015	0.903	0.955	<0.001
197	MFG.L	FPN	(-34, 55, 4)	0.046	0.830	0.917	0.001
198	ORBmid.L	FPN	(-42, 45, -2)	1.016	0.316	0.612	0.012
199	ANG.R	FPN	(33, -53, 44)	0.254	0.616	0.817	0.003
200	ORBinf.R	FPN	(43, 49, -2)	0.089	0.766	0.887	0.001
201	IFGtriang.L	FPN	(-42, 25, 30)	0.164	0.687	0.860	0.002
202	ORBmid.L	FPN	(-3, 26, 44)	3.551	0.063	0.313	0.040
203	DCG.R	SAL	(11, -39, 50)	0.999	0.320	0.612	0.012
204	SMG.R	SAL	(55, -45, 37)	0.266	0.607	0.817	0.003
205	PreCG.R	SAL	(42, 0, 47)	1.227	0.271	0.583	0.014
206	MFG.R	SAL	(31, 33, 26)	0.476	0.492	0.751	0.006
207	IFGtriang.R	SAL	(48, 22, 10)	0.002	0.969	0.984	<0.001
**208**	**INS.L**	**SAL**	**(-35, 20, 0)**	**12.862**	**0.001**	**0.049**	**0.131**

209	INS.R	SAL	(36, 22, 3)	4.878	0.030	0.268	0.054
210	ORBinf.R	SAL	(37, 32, -2)	0.007	0.932	0.969	<0.001
211	INS.R	SAL	(34, 16, -8)	0.280	0.598	0.817	0.003
212	ACG.L	SAL	(-11, 26, 25)	1.262	0.264	0.577	0.015
213	DCG.L	SAL	(-1, 15, 44)	0.205	0.652	0.843	0.002
214	MFG.L	SAL	(-28, 52, 21)	0.797	0.375	0.664	0.009
215	ACG.L	SAL	(0, 30, 27)	4.847	0.030	0.268	0.054
216	DCG.R	SAL	(5, 23, 37)	0.599	0.441	0.723	0.007
217	ACG.R	SAL	(10, 22, 27)	0.188	0.665	0.847	0.002
218	SFGdor.R	SAL	(31, 56, 14)	2.002	0.161	0.480	0.023
219	MFG.R	SAL	(26, 50, 27)	2.069	0.154	0.480	0.024
220	MFG.L	SAL	(-39, 51, 17)	0.020	0.888	0.955	<0.001
221	MFG.R	SAL	(2, -24, 30)	1.052	0.308	0.612	0.012
222	THA.R	SUB	(6, -24, 0)	1.092	0.299	0.612	0.013
223	THA.L	SUB	(-2, -13, 12)	0.716	0.400	0.681	0.008
224	THA.L	SUB	(-10, -18, 7)	2.256	0.137	0.463	0.026
225	THA.R	SUB	(12, -17, 8)	3.465	0.066	0.320	0.039
226	THA.L	SUB	(-5, -28, -4)	0.550	0.460	0.736	0.006
227	PUT.L	SUB	(-22, 7, -5)	2.321	0.131	0.450	0.027
228	CAU.L	SUB	(-15, 4, 8)	1.655	0.202	0.517	0.019
229	PUT.R	SUB	(31, -14, 2)	0.260	0.611	0.817	0.003
230	PUT.R	SUB	(23, 10, 1)	0.755	0.387	0.664	0.009
231	PUT.R	SUB	(29, 1, 4)	0.104	0.748	0.887	0.001
232	PUT.L	SUB	(-31, -11, 0)	0.226	0.635	0.826	0.003
233	CAU.R	SUB	(15, 5, 7)	1.928	0.169	0.480	0.022
234	THA.R	SUB	(9, -4, 6)	3.759	0.056	0.301	0.042
235	STG.R	VAN	(54, -43, 22)	1.580	0.212	0.520	0.018
236	MTG.L	VAN	(-56, -50, 10)	0.120	0.730	0.878	0.001
237	STG.L	VAN	(-55, -40, 14)	4.259	0.042	0.294	0.048
238	STG.R	VAN	(52, -33, 8)	0.136	0.714	0.868	0.002
239	MTG.R	VAN	(51, -29, -4)	7.790	0.006	0.156	0.084
240	MTG.R	VAN	(56, -46, 11)	8.871	0.004	0.118	0.095
241	IFGtriang.R	VAN	(53, 33, 1)	0.016	0.900	0.955	<0.001
242	IFGtriang.L	VAN	(-49, 25, -1)	1.435	0.234	0.528	0.017
243	No	No	(-16, -65, -20)	2.562	0.113	0.432	0.029
244	No	No	(-32, -55, -25)	4.581	0.035	0.273	0.051
245	No	No	(22, -58, -23)	5.874	0.017	0.229	0.065
246	No	No	(1, -62, -18)	4.097	0.046	0.297	0.046
247	No	No	(33, -12, -34)	0.267	0.607	0.817	0.003
248	No	No	(-31, -10, -36)	0.002	0.969	0.984	<0.001
249	No	No	(49, -3, -38)	5.324	0.023	0.248	0.059
250	No	No	(-50, -7, -39)	5.798	0.018	0.229	0.064
251	PCUN.R	DAN	(10, -62, 61)	0.030	0.863	0.938	<0.001

252	MTG.L	DAN	(-52，-63，5)	1.440	0.234	0.528	0.017
253	No	No	(-47，-51，-21)	1.983	0.163	0.480	0.023
254	No	No	(46，-47，-17)	1.007	0.318	0.612	0.012
255	IPL.R	MSN	(47，-30，49)	0.186	0.667	0.847	0.002
256	SOG.R	DAN	(22，-65，48)	0.151	0.698	0.861	0.002
257	MTG.R	DAN	(46，-59，4)	0.144	0.705	0.865	0.002
258	SPG.R	DAN	(25，-58，60)	1.951	0.166	0.480	0.022
259	IPL.L	DAN	(-33，-46，47)	6.300	0.014	0.211	0.069
260	SOG.L	DAN	(-27，-71，37)	0.001	0.977	0.989	<0.001
261	MFG.L	DAN	(-32，-1，54)	0.759	0.386	0.664	0.009
262	ITG.L	DAN	(-42，-60，-9)	0.300	0.586	0.816	0.004
263	SPG.L	DAN	(-17，-59，64)	0.014	0.905	0.955	<0.001
264	PreCG.R	DAN	(29，-5，54)	3.308	0.072	0.320	0.037

Table S2. Node entropy group differences at σ = 0.5 (BD vs. NCs). Regions showing statistically significant differences (FDR corrected $q < 0.05$) are marked in bold and red.

No.	ROI	Module	Peak MNI	F-value	p-value	q-value	η_p^2
1	No	No	(-25, -98, -12)	4.387	0.039	0.134	0.049
2	No	No	(27, -97, -13)	1.039	0.311	0.458	0.012
3	No	No	(24, 32, -18)	0.469	0.495	0.631	0.005
4	No	No	(-56, -45, -24)	0.229	0.633	0.732	0.003
5	No	No	(8, 41, -24)	0.492	0.485	0.627	0.006
6	No	No	(-21, -22, -20)	2.496	0.118	0.257	0.029
7	No	No	(17, -28, -17)	2.556	0.114	0.250	0.029
8	No	No	(-37, -29, -26)	0.134	0.715	0.796	0.002
9	No	No	(65, -24, -19)	9.604	0.003	0.032	0.102
10	No	No	(52, -34, -27)	1.355	0.248	0.401	0.016
11	No	No	(55, -31, -17)	0.702	0.404	0.556	0.008
12	No	No	(34, 38, -12)	0.081	0.777	0.847	0.001
13	PCUN.L	MSN	(-7, -52, 61)	4.251	0.042	0.137	0.048
14	DCG.L	MSN	(-14, -18, 40)	0.601	0.440	0.590	0.007
15	DCG.L	MSN	(0, -15, 47)	4.053	0.047	0.144	0.046
16	DCG.R	MSN	(10, -2, 45)	4.744	0.032	0.123	0.053
17	PCL.L	MSN	(-7, -21, 65)	0.494	0.484	0.627	0.006
18	PCL.L	MSN	(-7, -33, 72)	0.303	0.584	0.707	0.004
19	PoCG.R	MSN	(13, -33, 75)	6.873	0.010	0.070	0.075
**20**	**SMG.L**	**MSN**	**(-54, -23, 43)**	**12.730**	**0.001**	**0.028**	**0.130**
**21**	**PreCG.R**	**MSN**	**(29, -17, 71)**	**11.734**	**0.001**	**0.029**	**0.121**
22	PCUN.R	MSN	(10, -46, 73)	0.059	0.808	0.862	0.001
23	PoCG.L	MSN	(-23, -30, 72)	5.837	0.018	0.090	0.064
**24**	**PoCG.L**	**MSN**	**(-40, -19, 54)**	**10.558**	**0.002**	**0.031**	**0.110**
25	PoCG.R	MSN	(29, -39, 59)	4.021	0.048	0.144	0.045
26	PoCG.R	MSN	(50, -20, 42)	6.196	0.015	0.087	0.068
27	PoCG.L	MSN	(-38, -27, 69)	5.271	0.024	0.103	0.058
28	PreCG.R	MSN	(20, -29, 60)	0.534	0.467	0.610	0.006
**29**	**PreCG.R**	**MSN**	**(44, -8, 57)**	**11.141**	**0.001**	**0.030**	**0.116**
30	PoCG.L	MSN	(-29, -43, 61)	6.728	0.011	0.070	0.073
31	SMA.R	MSN	(10, -17, 74)	0.484	0.488	0.629	0.006
32	PoCG.R	MSN	(22, -42, 69)	3.500	0.065	0.178	0.040
**33**	**PoCG.L**	**MSN**	**(-45, -32, 47)**	**9.790**	**0.002**	**0.032**	**0.103**
34	PoCG.L	MSN	(-21, -31, 61)	5.655	0.020	0.091	0.062
35	PCL.L	MSN	(-13, -17, 75)	0.057	0.811	0.862	0.001
**36**	**PoCG.R**	**MSN**	**(42, -20, 55)**	**16.787**	**<0.001**	**0.012**	**0.165**
**37**	**PreCG.L**	**MSN**	**(-38, -15, 69)**	**8.283**	**0.005**	**0.045**	**0.089**
38	SPG.L	MSN	(-16, -46, 73)	<0.001	0.993	0.993	<0.001
39	PCL.R	MSN	(2, -28, 60)	2.227	0.139	0.290	0.026

40	SMA.R	MSN	(3, -17, 58)	2.854	0.095	0.220	0.032
**41**	**PreCG.R**	**MSN**	**(38, -17, 45)**	**9.458**	**0.003**	**0.032**	**0.100**
42	No	No	(-49, -11, 35)	5.422	0.022	0.100	0.060
43	No	No	(36, -9, 14)	1.977	0.163	0.317	0.023
44	No	No	(51, -6, 32)	4.372	0.040	0.134	0.049
45	No	No	(-53, -10, 24)	3.442	0.067	0.181	0.039
46	No	No	(66, -8, 25)	7.874	0.006	0.051	0.085
47	SMA.L	CON	(-3, 2, 53)	0.161	0.689	0.783	0.002
48	SMG.R	CON	(54, -28, 34)	2.973	0.088	0.209	0.034
49	SFGdor.R	CON	(19, -8, 64)	1.221	0.272	0.423	0.014
50	SFGdor.L	CON	(-16, -5, 71)	1.285	0.260	0.418	0.015
51	DCG.L	CON	(-10, -2, 42)	2.254	0.137	0.289	0.026
52	INS.R	CON	(37, 1, -4)	4.031	0.048	0.144	0.045
53	SMA.R	CON	(13, -1, 70)	0.047	0.829	0.863	0.001
54	SMA.R	CON	(7, 8, 51)	4.335	0.040	0.135	0.049
55	ROL.L	CON	(-45, 0, 9)	1.083	0.301	0.451	0.013
56	INS.R	CON	(49, 8, -1)	1.736	0.191	0.358	0.020
57	PUT.L	CON	(-34, 3, 4)	4.117	0.046	0.142	0.046
58	TPOsup.L	CON	(-51, 8, -2)	1.368	0.245	0.400	0.016
59	DCG.L	CON	(-5, 18, 34)	1.548	0.217	0.367	0.018
60	INS.R	CON	(36, 10, 1)	1.492	0.225	0.376	0.017
61	Heschl_R	AUD	(32, -26, 13)	2.701	0.104	0.235	0.031
62	STG.R	AUD	(65, -33, 20)	0.956	0.331	0.480	0.011
**63**	**STG.R**	**AUD**	**(58, -16, 7)**	**9.123**	**0.003**	**0.033**	**0.097**
64	ROL.L	AUD	(-38, -33, 17)	0.718	0.399	0.552	0.008
65	STG.L	AUD	(-60, -25, 14)	0.003	0.956	0.968	<0.001
66	STG.L	AUD	(-49, -26, 5)	5.690	0.019	0.091	0.063
67	ROL.R	AUD	(43, -23, 20)	7.041	0.010	0.069	0.076
68	SMG.L	AUD	(-50, -34, 26)	4.225	0.043	0.137	0.047
69	SMG.L	AUD	(-53, -22, 23)	0.095	0.759	0.831	0.001
70	HES.L	AUD	(-55, -9, 12)	3.195	0.077	0.189	0.036
71	ROL.R	AUD	(56, -5, 13)	0.424	0.517	0.646	0.005
72	SMG.R	AUD	(59, -17, 29)	1.368	0.245	0.400	0.016
73	ROL.L	AUD	(-30, -27, 12)	0.013	0.910	0.936	<0.001
74	MOG.L	DMN	(-41, -75, 26)	3.062	0.084	0.203	0.035
75	ORBmid.R	DMN	(6, 67, -4)	0.189	0.665	0.760	0.002
76	Rectus_R	DMN	(8, 48, -15)	0.693	0.408	0.558	0.008
77	LING.L	DMN	(-13, -40, 1)	1.060	0.306	0.454	0.012
78	ORBsup.L	DMN	(-18, 63, -9)	2.213	0.141	0.290	0.025
**79**	**MTG.L**	**DMN**	**(-46, -61, 21)**	**10.797**	**0.001**	**0.031**	**0.113**
**80**	**MOG.R**	**DMN**	**(43, -72, 28)**	**9.624**	**0.003**	**0.032**	**0.102**
81	TPOmid.L	DMN	(-44, 12, -34)	0.261	0.611	0.724	0.003
82	TPOmid.R	DMN	(46, 16, -30)	0.239	0.626	0.732	0.003

83	ITG.L	DMN	(-68, -23, -16)	2.351	0.129	0.279	0.027
84	No	No	(-58, -26, -15)	6.130	0.015	0.087	0.067
85	No	No	(27, 16, -17)	0.560	0.457	0.600	0.007
**86**	**ANG.L**	**DMN**	**(-44，-65，35)**	**10.543**	**0.002**	**0.031**	**0.110**
87	IPL.L	DMN	(-39, -75, 44)	6.804	0.011	0.070	0.074
**88**	**PCUN.L**	**DMN**	**(-7，-55，27)**	**9.742**	**0.002**	**0.032**	**0.103**
89	PCUN.R	DMN	(6, -59, 35)	0.012	0.915	0.936	<0.001
90	PCUN.L	DMN	(-11, -56, 16)	3.671	0.059	0.165	0.041
91	PCUN.L	DMN	(-3, -49, 13)	5.329	0.023	0.101	0.059
92	PCG.R	DMN	(8, -48, 31)	6.697	0.011	0.070	0.073
93	PCUN.R	DMN	(15, -63, 26)	1.650	0.202	0.359	0.019
**94**	**DCG.L**	**DMN**	**(-2，-37，44)**	**10.078**	**0.002**	**0.032**	**0.106**
**95**	**PCUN.R**	**DMN**	**(11，-54，17)**	**9.345**	**0.003**	**0.033**	**0.099**
**96**	**ANG.R**	**DMN**	**(52，-59，36)**	**9.237**	**0.003**	**0.033**	**0.098**
97	SFGdor.R	DMN	(23, 33, 48)	0.051	0.821	0.863	0.001
98	ORBmid.L	DMN	(-10, 39, 52)	1.093	0.299	0.451	0.013
99	SFGdor.L	DMN	(-16, 29, 53)	0.799	0.374	0.528	0.009
100	MFG.L	DMN	(-35, 20, 51)	2.747	0.101	0.230	0.031
101	SFGdor.R	DMN	(22, 39, 39)	0.046	0.830	0.863	0.001
102	SFGdor.R	DMN	(13, 55, 38)	5.046	0.027	0.111	0.056
103	SFGdor.L	DMN	(-10, 55, 39)	1.695	0.196	0.359	0.020
104	SFGdor.L	DMN	(-20, 45, 39)	0.782	0.379	0.531	0.009
105	SFGmed.R	DMN	(6, 54, 16)	0.867	0.354	0.505	0.010
106	SFGmed.R	DMN	(6, 64, 22)	0.420	0.518	0.646	0.005
107	ACG.L	DMN	(-7, 51, -1)	4.696	0.033	0.123	0.052
108	SFGmed.R	DMN	(9, 54, 3)	2.818	0.097	0.222	0.032
109	ORBmid.L	DMN	(-3, 44, -9)	4.573	0.035	0.127	0.051
110	ORBmid.R	DMN	(8, 42, -5)	7.009	0.010	0.069	0.076
111	ACG.L	DMN	(-11, 45, 8)	0.003	0.958	0.968	<0.001
**112**	**ORBmid.L**	**DMN**	**(-2，38，36)**	**8.641**	**0.004**	**0.039**	**0.092**
**113**	**ACG.L**	**DMN**	**(-3，42，16)**	**15.389**	**<0.001**	**0.012**	**0.153**
114	SFGdor.L	DMN	(-20, 64, 19)	0.431	0.513	0.645	0.005
115	ORBmid.L	DMN	(-8, 48, 23)	3.901	0.051	0.149	0.044
116	MTG.R	DMN	(65, -12, -19)	1.243	0.268	0.419	0.014
117	MTG.L	DMN	(-56, -13, -10)	5.881	0.017	0.090	0.065
118	MTG.L	DMN	(-58, -30, -4)	6.254	0.014	0.086	0.069
119	MTG.R	DMN	(65, -31, -9)	0.466	0.497	0.631	0.005
120	MTG.L	DMN	(-68, -41, -5)	0.433	0.512	0.645	0.005
121	SFGdor.R	DMN	(13, 30, 59)	2.187	0.143	0.290	0.025
**122**	**ACG.R**	**DMN**	**(12，36，20)**	**10.460**	**0.002**	**0.031**	**0.110**
123	MTG.R	DMN	(52, -2, -16)	3.283	0.074	0.187	0.037
124	PHG.L	DMN	(-26, -40, -8)	5.943	0.017	0.090	0.065
125	FFG.R	DMN	(27, -37, -13)	4.630	0.034	0.126	0.052

126	FFG.L	DMN	(-34, -38, -16)	3.401	0.069	0.183	0.038
127	Cerebelum	DMN	(28, -77, -32)	0.158	0.692	0.783	0.002
128	TPOmid.R	DMN	(52, 7, -30)	1.664	0.201	0.359	0.019
129	MTG.L	DMN	(-53, 3, -27)	1.258	0.265	0.418	0.015
**130**	**ANG.R**	**DMN**	**(47, -50, 29)**	**15.697**	**<0.001**	**0.012**	**0.156**
131	MTG.L	DMN	(-49, -42, 1)	1.521	0.221	0.371	0.018
132	No	No	(-31, 19, -19)	0.474	0.493	0.631	0.006
133	No	No	(-2, -35, 31)	3.732	0.057	0.163	0.042
134	No	No	(-7, -71, 42)	6.108	0.015	0.087	0.067
135	No	No	(11, -66, 42)	3.488	0.065	0.178	0.039
136	No	No	(4, -48, 51)	1.026	0.314	0.458	0.012
137	ORBinf.L	DMN	(-46, 31, -13)	0.097	0.756	0.831	0.001
138	SMA.L	VAN	(-10, 11, 67)	0.122	0.728	0.807	0.001
139	ORBinf.R	DMN	(49, 35, -12)	3.233	0.076	0.188	0.037
140	No	No	(8, -91, -7)	0.002	0.966	0.970	<0.001
141	No	No	(17, -91, -14)	2.966	0.089	0.209	0.034
142	No	No	(-12, -95, -13)	7.445	0.008	0.062	0.081
143	LING.R	VIS	(18, -47, -10)	1.156	0.285	0.435	0.013
144	MOG.R	VIS	(40, -72, 14)	7.087	0.009	0.069	0.077
145	CAL.R	VIS	(8, -72, 11)	0.895	0.347	0.497	0.010
146	CAL.L	VIS	(-8, -81, 7)	0.277	0.600	0.720	0.003
147	MOG.L	VIS	(-28, -79, 19)	0.061	0.805	0.862	0.001
148	LING.R	VIS	(20, -66, 2)	0.025	0.875	0.906	<0.001
149	MOG.L	VIS	(-24, -91, 19)	2.024	0.159	0.312	0.023
150	FFG.R	VIS	(27, -59, -9)	4.214	0.043	0.137	0.047
151	LING.L	VIS	(-15, -72, -8)	0.565	0.454	0.600	0.007
152	CAL.L	VIS	(-18, -68, 5)	0.237	0.628	0.732	0.003
153	IOG.R	VIS	(43, -78, -12)	5.909	0.017	0.090	0.065
154	IOG.L	VIS	(-47, -76, -10)	5.751	0.019	0.090	0.063
155	SOG.L	VIS	(-14, -91, 31)	1.078	0.302	0.451	0.013
156	SOG.R	VIS	(15, -87, 37)	1.264	0.264	0.418	0.015
157	MOG.R	VIS	(29, -77, 25)	0.074	0.786	0.850	0.001
158	LING.R	VIS	(20, -86, -2)	0.049	0.825	0.863	0.001
159	CUN.R	VIS	(15, -77, 31)	0.002	0.961	0.968	<0.001
160	LING.L	VIS	(-16, -52, -1)	1.033	0.312	0.458	0.012
161	ITG.R	VIS	(42, -66, -8)	1.868	0.175	0.335	0.022
162	SOG.R	VIS	(24, -87, 24)	0.320	0.573	0.700	0.004
163	CUN.R	VIS	(6, -72, 24)	0.779	0.380	0.531	0.009
**164**	**MOG.L**	**VIS**	**(-42, -74, 0)**	**9.683**	**0.003**	**0.032**	**0.102**
165	Cerebelum	VIS	(26, -79, -16)	1.651	0.202	0.359	0.019
166	CUN.L	VIS	(-16, -77, 34)	0.051	0.822	0.863	0.001
167	CUN.L	VIS	(-3, -81, 21)	0.004	0.949	0.968	<0.001
168	MOG.L	VIS	(-40, -88, -6)	2.259	0.137	0.289	0.026

169	MOG.R	VIS	(37, -84, 13)	4.542	0.036	0.127	0.051
170	CAL.R	VIS	(6, -81, 6)	0.142	0.707	0.795	0.002
171	MOG.L	VIS	(-26, -90, 3)	2.009	0.160	0.313	0.023
172	FFG.L	VIS	(-33, -79, -13)	2.199	0.142	0.290	0.025
173	MOG.R	VIS	(37, -81, 1)	4.771	0.032	0.123	0.053
174	PreCG.L	FPN	(-44, 2, 46)	2.233	0.139	0.290	0.026
175	IFGtriang.R	FPN	(48, 25, 27)	0.117	0.733	0.810	0.001
176	ORBinf.L	FPN	(-47, 11, 23)	0.056	0.813	0.862	0.001
**177**	**IPL.L**	**FPN**	**(-53, -49, 43)**	**11.432**	**0.001**	**0.029**	**0.119**
178	MFG.L	FPN	(-23, 11, 64)	0.722	0.398	0.552	0.008
179	ITG.R	FPN	(58, -53, -14)	3.388	0.069	0.183	0.038
180	ORBsup.R	FPN	(24, 45, -15)	0.586	0.446	0.595	0.007
181	ORBmid.R	FPN	(34, 54, -13)	1.930	0.168	0.325	0.022
182	No	No	(-21, 41, -20)	0.298	0.587	0.707	0.003
183	No	No	(-18, -76, -24)	0.137	0.712	0.796	0.002
184	No	No	(17, -80, -34)	1.395	0.241	0.397	0.016
185	No	No	(35, -67, -34)	1.267	0.263	0.418	0.015
186	PreCG.R	FPN	(47, 10, 33)	3.282	0.074	0.187	0.037
187	PreCG.L	FPN	(-41, 6, 33)	0.404	0.527	0.653	0.005
188	MFG.L	FPN	(-42, 38, 21)	3.960	0.050	0.148	0.045
189	MFG.R	FPN	(38, 43, 15)	2.072	0.154	0.307	0.024
190	SMG.R	FPN	(49, -42, 45)	1.615	0.207	0.360	0.019
191	SPG.L	FPN	(-28, -58, 48)	1.674	0.199	0.359	0.019
192	IPL.R	FPN	(44, -53, 47)	5.115	0.026	0.108	0.057
193	MFG.R	FPN	(32, 14, 56)	1.715	0.194	0.359	0.020
194	ANG.R	FPN	(37, -65, 40)	1.603	0.209	0.361	0.019
**195**	**ANG.L**	**FPN**	**(-42, -55, 45)**	**11.851**	**0.001**	**0.029**	**0.122**
196	MFG.R	FPN	(40, 18, 40)	3.715	0.057	0.163	0.042
197	MFG.L	FPN	(-34, 55, 4)	0.155	0.694	0.783	0.002
198	ORBmid.L	FPN	(-42, 45, -2)	2.162	0.145	0.292	0.025
199	ANG.R	FPN	(33, -53, 44)	3.579	0.062	0.172	0.040
200	ORBinf.R	FPN	(43, 49, -2)	0.378	0.540	0.666	0.004
201	IFGtriang.L	FPN	(-42, 25, 30)	0.227	0.635	0.732	0.003
202	ORBmid.L	FPN	(-3, 26, 44)	6.728	0.011	0.070	0.073
203	DCG.R	SAL	(11, -39, 50)	1.701	0.196	0.359	0.020
204	SMG.R	SAL	(55, -45, 37)	2.965	0.089	0.209	0.034
205	PreCG.R	SAL	(42, 0, 47)	0.339	0.562	0.690	0.004
206	MFG.R	SAL	(31, 33, 26)	0.260	0.611	0.724	0.003
207	IFGtriang.R	SAL	(48, 22, 10)	0.613	0.436	0.590	0.007
**208**	**INS.L**	**SAL**	**(-35, 20, 0)**	**19.712**	**<0.001**	**0.007**	**0.188**
209	INS.R	SAL	(36, 22, 3)	5.739	0.019	0.090	0.063
210	ORBinf.R	SAL	(37, 32, -2)	0.605	0.439	0.590	0.007
211	INS.R	SAL	(34, 16, -8)	0.233	0.631	0.732	0.003

212	ACG.L	SAL	(-11, 26, 25)	1.561	0.215	0.367	0.018
213	DCG.L	SAL	(-1, 15, 44)	0.579	0.449	0.596	0.007
214	MFG.L	SAL	(-28, 52, 21)	4.185	0.044	0.138	0.047
**215**	**ACG.L**	**SAL**	**(0, 30, 27)**	**10.245**	**0.002**	**0.032**	**0.108**
216	DCG.R	SAL	(5, 23, 37)	4.533	0.036	0.127	0.051
217	ACG.R	SAL	(10, 22, 27)	0.612	0.436	0.590	0.007
218	SFGdor.R	SAL	(31, 56, 14)	2.287	0.134	0.288	0.026
219	MFG.R	SAL	(26, 50, 27)	3.344	0.071	0.185	0.038
220	MFG.L	SAL	(-39, 51, 17)	1.672	0.199	0.359	0.019
**221**	**MFG.R**	**SAL**	**(2, -24, 30)**	**9.108**	**0.003**	**0.033**	**0.097**
222	THA.R	SUB	(6, -24, 0)	1.557	0.216	0.367	0.018
223	THA.L	SUB	(-2, -13, 12)	3.228	0.076	0.188	0.037
224	THA.L	SUB	(-10, -18, 7)	4.902	0.030	0.118	0.055
225	THA.R	SUB	(12, -17, 8)	4.694	0.033	0.123	0.052
226	THA.L	SUB	(-5, -28, -4)	5.178	0.025	0.106	0.057
227	PUT.L	SUB	(-22, 7, -5)	2.678	0.105	0.235	0.031
228	CAU.L	SUB	(-15, 4, 8)	4.247	0.042	0.137	0.048
229	PUT.R	SUB	(31, -14, 2)	0.079	0.780	0.847	0.001
230	PUT.R	SUB	(23, 10, 1)	1.198	0.277	0.425	0.014
231	PUT.R	SUB	(29, 1, 4)	0.251	0.617	0.728	0.003
232	PUT.L	SUB	(-31, -11, 0)	0.310	0.579	0.704	0.004
233	CAU.R	SUB	(15, 5, 7)	1.819	0.181	0.341	0.021
234	THA.R	SUB	(9, -4, 6)	6.948	0.010	0.069	0.076
235	STG.R	VAN	(54, -43, 22)	1.476	0.228	0.378	0.017
236	MTG.L	VAN	(-56, -50, 10)	3.291	0.073	0.187	0.037
237	STG.L	VAN	(-55, -40, 14)	5.852	0.018	0.090	0.064
238	STG.R	VAN	(52, -33, 8)	2.887	0.093	0.217	0.033
**239**	**MTG.R**	**VAN**	**(51, -29, -4)**	**8.064**	**0.006**	**0.048**	**0.087**
240	MTG.R	VAN	(56, -46, 11)	6.978	0.010	0.069	0.076
241	IFGtriang.R	VAN	(53, 33, 1)	0.012	0.913	0.936	<0.001
242	IFGtriang.L	VAN	(-49, 25, -1)	1.819	0.181	0.341	0.021
243	No	No	(-16, -65, -20)	1.634	0.205	0.360	0.019
244	No	No	(-32, -55, -25)	5.425	0.022	0.100	0.060
245	No	No	(22, -58, -23)	8.633	0.004	0.039	0.092
246	No	No	(1, -62, -18)	4.809	0.031	0.122	0.054
247	No	No	(33, -12, -34)	0.263	0.610	0.724	0.003
248	No	No	(-31, -10, -36)	1.619	0.207	0.360	0.019
249	No	No	(49, -3, -38)	2.672	0.106	0.235	0.030
250	No	No	(-50, -7, -39)	5.350	0.023	0.101	0.059
251	PCUN.R	DAN	(10, -62, 61)	0.193	0.661	0.759	0.002
252	MTG.L	DAN	(-52, -63, 5)	5.778	0.018	0.090	0.064
253	No	No	(-47, -51, -21)	0.067	0.796	0.858	0.001
254	No	No	(46, -47, -17)	4.370	0.040	0.134	0.049

255	IPL.R	MSN	（47， -30， 49）	1.096	0.298	0.451	0.013
256	SOG.R	DAN	（22， -65， 48）	1.200	0.276	0.425	0.014
257	MTG.R	DAN	（46， -59， 4）	3.226	0.076	0.188	0.037
258	SPG.R	DAN	（25， -58， 60）	3.910	0.051	0.149	0.044
**259**	**IPL.L**	**DAN**	**（-33，-46，47）**	**12.613**	**0.001**	**0.028**	**0.129**
260	SOG.L	DAN	（-27， -71， 37）	0.862	0.356	0.505	0.010
261	MFG.L	DAN	（-32， -1， 54）	0.905	0.344	0.496	0.011
262	ITG.L	DAN	（-42， -60， -9）	1.253	0.266	0.418	0.015
263	SPG.L	DAN	（-17， -59， 64）	2.044	0.157	0.311	0.023
**264**	**PreCG.R**	**DAN**	**（29，-5，54）**	**11.454**	**0.001**	**0.029**	**0.119**

Table S3. Node entropy group differences at σ = 0.7 (BD vs. NCs). Regions showing statistically significant differences (FDR corrected $q < 0.05$) are marked in bold and red.

No.	ROI	Module	Peak MNI	*F*-value	*p*-value	*q*-value	η_p^2
1	No	No	(-25, -98, -12)	5.738	0.019	0.083	0.063
2	No	No	(27, -97, -13)	2.289	0.134	0.251	0.026
3	No	No	(24, 32, -18)	0.222	0.639	0.697	0.003
4	No	No	(-56, -45, -24)	0.132	0.718	0.767	0.002
5	No	No	(8, 41, -24)	0.192	0.663	0.717	0.002
6	No	No	(-21, -22, -20)	3.372	0.070	0.172	0.038
7	No	No	(17, -28, -17)	3.609	0.061	0.154	0.041
8	No	No	(-37, -29, -26)	0.020	0.889	0.918	<0.001
9	No	No	(65, -24, -19)	11.444	0.001	0.030	0.119
10	No	No	(52, -34, -27)	1.798	0.184	0.305	0.021
11	No	No	(55, -31, -17)	0.827	0.366	0.485	0.010
12	No	No	(34, 38, -12)	0.007	0.934	0.948	<0.001
13	PCUN.L	MSN	(-7, -52, 61)	4.667	0.034	0.108	0.052
14	DCG.L	MSN	(-14, -18, 40)	0.583	0.447	0.549	0.007
15	DCG.L	MSN	(0, -15, 47)	4.404	0.039	0.120	0.049
16	DCG.R	MSN	(10, -2, 45)	3.890	0.052	0.145	0.044
17	PCL.L	MSN	(-7, -21, 65)	0.661	0.418	0.531	0.008
18	PCL.L	MSN	(-7, -33, 72)	0.872	0.353	0.478	0.010
19	PoCG.R	MSN	(13, -33, 75)	7.825	0.006	0.053	0.084
**20**	**SMG.L**	**MSN**	**(-54, -23, 43)**	**9.403**	**0.003**	**0.035**	**0.100**
**21**	**PreCG.R**	**MSN**	**(29, -17, 71)**	**12.934**	**0.001**	**0.024**	**0.132**
22	PCUN.R	MSN	(10, -46, 73)	0.605	0.439	0.543	0.007
23	PoCG.L	MSN	(-23, -30, 72)	6.655	0.012	0.071	0.073
**24**	**PoCG.L**	**MSN**	**(-40, -19, 54)**	**8.471**	**0.005**	**0.049**	**0.091**
25	PoCG.R	MSN	(29, -39, 59)	4.928	0.029	0.102	0.055
26	PoCG.R	MSN	(50, -20, 42)	5.656	0.020	0.085	0.062
27	PoCG.L	MSN	(-38, -27, 69)	5.097	0.027	0.097	0.057
28	PreCG.R	MSN	(20, -29, 60)	0.428	0.515	0.610	0.005
**29**	**PreCG.R**	**MSN**	**(44, -8, 57)**	**11.052**	**0.001**	**0.030**	**0.115**
30	PoCG.L	MSN	(-29, -43, 61)	7.472	0.008	0.056	0.081
31	SMA.R	MSN	(10, -17, 74)	1.960	0.165	0.283	0.023
32	PoCG.R	MSN	(22, -42, 69)	5.462	0.022	0.088	0.060
**33**	**PoCG.L**	**MSN**	**(-45, -32, 47)**	**8.194**	**0.005**	**0.050**	**0.088**
34	PoCG.L	MSN	(-21, -31, 61)	4.973	0.028	0.102	0.055
35	PCL.L	MSN	(-13, -17, 75)	0.953	0.332	0.456	0.011
**36**	**PoCG.R**	**MSN**	**(42, -20, 55)**	**16.456**	**<0.001**	**0.010**	**0.162**
37	PreCG.L	MSN	(-38, -15, 69)	6.191	0.015	0.077	0.068
38	SPG.L	MSN	(-16, -46, 73)	0.440	0.509	0.608	0.005
39	PCL.R	MSN	(2, -28, 60)	2.878	0.093	0.202	0.033

40	SMA.R	MSN	(3, -17, 58)	3.279	0.074	0.179	0.037
41	PreCG.R	MSN	(38, -17, 45)	7.420	0.008	0.056	0.080
42	No	No	(-49, -11, 35)	4.479	0.037	0.118	0.050
43	No	No	(36, -9, 14)	2.060	0.155	0.267	0.024
44	No	No	(51, -6, 32)	3.662	0.059	0.154	0.041
45	No	No	(-53, -10, 24)	2.591	0.111	0.219	0.030
46	No	No	(66, -8, 25)	8.277	0.005	0.050	0.089
47	SMA.L	CON	(-3, 2, 53)	0.242	0.624	0.690	0.003
48	SMG.R	CON	(54, -28, 34)	3.683	0.058	0.154	0.042
49	SFGdor.R	CON	(19, -8, 64)	1.255	0.266	0.390	0.015
50	SFGdor.L	CON	(-16, -5, 71)	1.803	0.183	0.305	0.021
51	DCG.L	CON	(-10, -2, 42)	2.214	0.140	0.254	0.025
52	INS.R	CON	(37, 1, -4)	3.853	0.053	0.146	0.043
53	SMA.R	CON	(13, -1, 70)	0.147	0.702	0.754	0.002
54	SMA.R	CON	(7, 8, 51)	4.670	0.034	0.108	0.052
55	ROL.L	CON	(-45, 0, 9)	1.356	0.247	0.369	0.016
56	INS.R	CON	(49, 8, -1)	2.110	0.150	0.265	0.024
57	PUT.L	CON	(-34, 3, 4)	3.646	0.060	0.154	0.041
58	TPOsup.L	CON	(-51, 8, -2)	1.684	0.198	0.319	0.019
59	DCG.L	CON	(-5, 18, 34)	1.050	0.308	0.431	0.012
60	INS.R	CON	(36, 10, 1)	2.093	0.152	0.265	0.024
61	Heschl_R	AUD	(32, -26, 13)	2.399	0.125	0.241	0.027
62	STG.R	AUD	(65, -33, 20)	1.506	0.223	0.348	0.017
**63**	**STG.R**	**AUD**	**(58, -16, 7)**	**9.433**	**0.003**	**0.035**	**0.100**
64	ROL.L	AUD	(-38, -33, 17)	0.625	0.431	0.540	0.007
65	STG.L	AUD	(-60, -25, 14)	0.007	0.932	0.948	<0.001
66	STG.L	AUD	(-49, -26, 5)	5.157	0.026	0.096	0.057
67	ROL.R	AUD	(43, -23, 20)	6.310	0.014	0.073	0.069
68	SMG.L	AUD	(-50, -34, 26)	3.876	0.052	0.145	0.044
69	SMG.L	AUD	(-53, -22, 23)	0.485	0.488	0.589	0.006
70	HES.L	AUD	(-55, -9, 12)	2.785	0.099	0.210	0.032
71	ROL.R	AUD	(56, -5, 13)	0.212	0.646	0.702	0.002
72	SMG.R	AUD	(59, -17, 29)	2.097	0.151	0.265	0.024
73	ROL.L	AUD	(-30, -27, 12)	0.008	0.927	0.948	<0.001
74	MOG.L	DMN	(-41, -75, 26)	3.328	0.072	0.175	0.038
75	ORBmid.R	DMN	(6, 67, -4)	0.739	0.392	0.509	0.009
76	Rectus_R	DMN	(8, 48, -15)	0.716	0.400	0.515	0.008
77	LING.L	DMN	(-13, -40, 1)	1.197	0.277	0.402	0.014
78	ORBsup.L	DMN	(-18, 63, -9)	2.386	0.126	0.241	0.027
**79**	**MTG.L**	**DMN**	**(-46, -61, 21)**	**8.185**	**0.005**	**0.050**	**0.088**
**80**	**MOG.R**	**DMN**	**(43, -72, 28)**	**10.358**	**0.002**	**0.030**	**0.109**
81	TPOmid.L	DMN	(-44, 12, -34)	0.236	0.629	0.691	0.003
82	TPOmid.R	DMN	(46, 16, -30)	0.127	0.723	0.767	0.001

83	ITG.L	DMN	(-68, -23, -16)	2.569	0.113	0.220	0.029
84	No	No	(-58, -26, -15)	5.636	0.020	0.085	0.062
85	No	No	(27, 16, -17)	0.905	0.344	0.471	0.011
**86**	**ANG.L**	**DMN**	**(-44, -65, 35)**	**9.156**	**0.003**	**0.038**	**0.097**
87	IPL.L	DMN	(-39, -75, 44)	7.711	0.007	0.054	0.083
**88**	**PCUN.L**	**DMN**	**(-7, -55, 27)**	**9.453**	**0.003**	**0.035**	**0.100**
89	PCUN.R	DMN	(6, -59, 35)	0.049	0.825	0.861	0.001
90	PCUN.L	DMN	(-11, -56, 16)	2.077	0.153	0.266	0.024
91	PCUN.L	DMN	(-3, -49, 13)	4.874	0.030	0.104	0.054
92	PCG.R	DMN	(8, -48, 31)	6.481	0.013	0.072	0.071
93	PCUN.R	DMN	(15, -63, 26)	1.501	0.224	0.348	0.017
**94**	**DCG.L**	**DMN**	**(-2, -37, 44)**	**10.680**	**0.002**	**0.030**	**0.112**
**95**	**PCUN.R**	**DMN**	**(11, -54, 17)**	**8.131**	**0.005**	**0.050**	**0.087**
**96**	**ANG.R**	**DMN**	**(52, -59, 36)**	**10.877**	**0.001**	**0.030**	**0.113**
97	SFGdor.R	DMN	(23, 33, 48)	0.313	0.578	0.654	0.004
98	ORBmid.L	DMN	(-10, 39, 52)	1.713	0.194	0.316	0.020
99	SFGdor.L	DMN	(-16, 29, 53)	1.721	0.193	0.316	0.020
100	MFG.L	DMN	(-35, 20, 51)	2.605	0.110	0.219	0.030
101	SFGdor.R	DMN	(22, 39, 39)	0.123	0.726	0.767	0.001
102	SFGdor.R	DMN	(13, 55, 38)	4.832	0.031	0.105	0.054
103	SFGdor.L	DMN	(-10, 55, 39)	1.867	0.175	0.295	0.021
104	SFGdor.L	DMN	(-20, 45, 39)	0.879	0.351	0.478	0.010
105	SFGmed.R	DMN	(6, 54, 16)	0.458	0.500	0.600	0.005
106	SFGmed.R	DMN	(6, 64, 22)	0.736	0.393	0.509	0.009
107	ACG.L	DMN	(-7, 51, -1)	3.965	0.050	0.142	0.045
108	SFGmed.R	DMN	(9, 54, 3)	3.236	0.076	0.181	0.037
109	ORBmid.L	DMN	(-3, 44, -9)	4.089	0.046	0.134	0.046
110	ORBmid.R	DMN	(8, 42, -5)	6.397	0.013	0.072	0.070
111	ACG.L	DMN	(-11, 45, 8)	0.028	0.868	0.903	<0.001
**112**	**ORBmid.L**	**DMN**	**(-2, 38, 36)**	**9.906**	**0.002**	**0.033**	**0.104**
**113**	**ACG.L**	**DMN**	**(-3, 42, 16)**	**13.343**	**<0.001**	**0.024**	**0.136**
114	SFGdor.L	DMN	(-20, 64, 19)	1.074	0.303	0.428	0.012
115	ORBmid.L	DMN	(-8, 48, 23)	5.370	0.023	0.092	0.059
116	MTG.R	DMN	(65, -12, -19)	1.519	0.221	0.348	0.018
117	MTG.L	DMN	(-56, -13, -10)	7.025	0.010	0.065	0.076
118	MTG.L	DMN	(-58, -30, -4)	6.717	0.011	0.071	0.073
119	MTG.R	DMN	(65, -31, -9)	0.771	0.382	0.500	0.009
120	MTG.L	DMN	(-68, -41, -5)	0.364	0.548	0.626	0.004
121	SFGdor.R	DMN	(13, 30, 59)	2.876	0.094	0.202	0.033
122	ACG.R	DMN	(12, 36, 20)	10.243	0.002	0.030	0.108
123	MTG.R	DMN	(52, -2, -16)	3.756	0.056	0.150	0.042
124	PHG.L	DMN	(-26, -40, -8)	5.756	0.019	0.083	0.063
125	FFG.R	DMN	(27, -37, -13)	5.463	0.022	0.088	0.060

126	FFG.L	DMN	(-34, -38, -16)	3.452	0.067	0.166	0.039
127	Cerebelum	DMN	(28, -77, -32)	0.996	0.321	0.446	0.012
128	TPOmid.R	DMN	(52, 7, -30)	1.176	0.281	0.404	0.014
129	MTG.L	DMN	(-53, 3, -27)	1.656	0.202	0.323	0.019
**130**	**ANG.R**	**DMN**	**(47, -50, 29)**	**19.259**	**<0.001**	**0.004**	**0.185**
131	MTG.L	DMN	(-49, -42, 1)	1.925	0.169	0.286	0.022
132	No	No	(-31, 19, -19)	1.059	0.306	0.430	0.012
133	No	No	(-2, -35, 31)	5.155	0.026	0.096	0.057
134	No	No	(-7, -71, 42)	6.965	0.010	0.065	0.076
135	No	No	(11, -66, 42)	4.726	0.032	0.108	0.053
136	No	No	(4, -48, 51)	1.222	0.272	0.397	0.014
137	ORBinf.L	DMN	(-46, 31, -13)	0.004	0.951	0.959	<0.001
138	SMA.L	VAN	(-10, 11, 67)	0.003	0.958	0.962	<0.001
139	ORBinf.R	DMN	(49, 35, -12)	2.690	0.105	0.214	0.031
140	No	No	(8, -91, -7)	0.004	0.948	0.959	<0.001
141	No	No	(17, -91, -14)	3.021	0.086	0.197	0.034
142	No	No	(-12, -95, -13)	9.573	0.003	0.035	0.101
143	LING.R	VIS	(18, -47, -10)	1.502	0.224	0.348	0.017
144	MOG.R	VIS	(40, -72, 14)	7.815	0.006	0.053	0.084
145	CAL.R	VIS	(8, -72, 11)	0.684	0.411	0.526	0.008
146	CAL.L	VIS	(-8, -81, 7)	0.383	0.538	0.620	0.004
147	MOG.L	VIS	(-28, -79, 19)	0.395	0.532	0.619	0.005
148	LING.R	VIS	(20, -66, 2)	0.093	0.761	0.801	0.001
149	MOG.L	VIS	(-24, -91, 19)	2.763	0.100	0.210	0.031
150	FFG.R	VIS	(27, -59, -9)	4.434	0.038	0.120	0.050
151	LING.L	VIS	(-15, -72, -8)	1.086	0.300	0.426	0.013
152	CAL.L	VIS	(-18, -68, 5)	0.564	0.455	0.556	0.007
153	IOG.R	VIS	(43, -78, -12)	6.560	0.012	0.072	0.072
154	IOG.L	VIS	(-47, -76, -10)	6.419	0.013	0.072	0.070
155	SOG.L	VIS	(-14, -91, 31)	1.444	0.233	0.355	0.017
156	SOG.R	VIS	(15, -87, 37)	2.094	0.152	0.265	0.024
157	MOG.R	VIS	(29, -77, 25)	0.675	0.414	0.527	0.008
158	LING.R	VIS	(20, -86, -2)	0.018	0.893	0.918	<0.001
159	CUN.R	VIS	(15, -77, 31)	0.255	0.615	0.685	0.003
160	LING.L	VIS	(-16, -52, -1)	1.420	0.237	0.359	0.016
161	ITG.R	VIS	(42, -66, -8)	2.894	0.093	0.202	0.033
162	SOG.R	VIS	(24, -87, 24)	0.394	0.532	0.619	0.005
163	CUN.R	VIS	(6, -72, 24)	1.356	0.247	0.369	0.016
**164**	**MOG.L**	**VIS**	**(-42, -74, 0)**	**8.768**	**0.004**	**0.044**	**0.094**
165	Cerebelum	VIS	(26, -79, -16)	2.673	0.106	0.215	0.030
166	CUN.L	VIS	(-16, -77, 34)	0.403	0.527	0.619	0.005
167	CUN.L	VIS	(-3, -81, 21)	<0.001	0.984	0.984	<0.001
168	MOG.L	VIS	(-40, -88, -6)	2.891	0.093	0.202	0.033

169	MOG.R	VIS	(37, -84, 13)	6.438	0.013	0.072	0.070
170	CAL.R	VIS	(6, -81, 6)	0.167	0.684	0.737	0.002
171	MOG.L	VIS	(-26, -90, 3)	1.686	0.198	0.319	0.019
172	FFG.L	VIS	(-33, -79, -13)	2.773	0.100	0.210	0.032
173	MOG.R	VIS	(37, -81, 1)	5.931	0.017	0.081	0.065
174	PreCG.L	FPN	(-44, 2, 46)	2.723	0.103	0.212	0.031
175	IFGtriang.R	FPN	(48, 25, 27)	0.790	0.376	0.494	0.009
176	ORBinf.L	FPN	(-47, 11, 23)	0.329	0.568	0.646	0.004
**177**	**IPL.L**	**FPN**	**(-53, -49, 43)**	**11.530**	**0.001**	**0.030**	**0.119**
178	MFG.L	FPN	(-23, 11, 64)	1.262	0.265	0.390	0.015
179	ITG.R	FPN	(58, -53, -14)	3.793	0.055	0.149	0.043
180	ORBsup.R	FPN	(24, 45, -15)	0.250	0.618	0.686	0.003
181	ORBmid.R	FPN	(34, 54, -13)	2.226	0.139	0.254	0.026
182	No	No	(-21, 41, -20)	0.483	0.489	0.589	0.006
183	No	No	(-18, -76, -24)	0.394	0.532	0.619	0.005
184	No	No	(17, -80, -34)	2.759	0.100	0.210	0.031
185	No	No	(35, -67, -34)	2.227	0.139	0.254	0.026
186	PreCG.R	FPN	(47, 10, 33)	3.129	0.080	0.188	0.036
187	PreCG.L	FPN	(-41, 6, 33)	0.601	0.440	0.543	0.007
188	MFG.L	FPN	(-42, 38, 21)	5.835	0.018	0.083	0.064
189	MFG.R	FPN	(38, 43, 15)	2.219	0.140	0.254	0.025
190	SMG.R	FPN	(49, -42, 45)	1.932	0.168	0.286	0.022
191	SPG.L	FPN	(-28, -58, 48)	3.166	0.079	0.187	0.036
192	IPL.R	FPN	(44, -53, 47)	7.541	0.007	0.056	0.081
193	MFG.R	FPN	(32, 14, 56)	2.310	0.132	0.249	0.026
194	ANG.R	FPN	(37, -65, 40)	3.026	0.086	0.197	0.034
**195**	**ANG.L**	**FPN**	**(-42, -55, 45)**	**13.791**	**<0.001**	**0.024**	**0.140**
196	MFG.R	FPN	(40, 18, 40)	4.714	0.033	0.108	0.053
197	MFG.L	FPN	(-34, 55, 4)	0.232	0.631	0.691	0.003
198	ORBmid.L	FPN	(-42, 45, -2)	2.423	0.123	0.239	0.028
199	ANG.R	FPN	(33, -53, 44)	4.929	0.029	0.102	0.055
200	ORBinf.R	FPN	(43, 49, -2)	0.828	0.365	0.485	0.010
201	IFGtriang.L	FPN	(-42, 25, 30)	0.495	0.484	0.589	0.006
202	ORBmid.L	FPN	(-3, 26, 44)	7.466	0.008	0.056	0.081
203	DCG.R	SAL	(11, -39, 50)	2.622	0.109	0.219	0.030
204	SMG.R	SAL	(55, -45, 37)	4.201	0.043	0.130	0.047
205	PreCG.R	SAL	(42, 0, 47)	0.292	0.590	0.666	0.003
206	MFG.R	SAL	(31, 33, 26)	0.272	0.603	0.678	0.003
207	IFGtriang.R	SAL	(48, 22, 10)	0.984	0.324	0.448	0.011
**208**	**INS.L**	**SAL**	**(-35, 20, 0)**	**20.612**	**<0.001**	**0.004**	**0.195**
209	INS.R	SAL	(36, 22, 3)	5.238	0.025	0.095	0.058
210	ORBinf.R	SAL	(37, 32, -2)	0.636	0.427	0.537	0.007
211	INS.R	SAL	(34, 16, -8)	0.370	0.544	0.625	0.004

212	ACG.L	SAL	(-11, 26, 25)	1.188	0.279	0.402	0.014
213	DCG.L	SAL	(-1, 15, 44)	0.857	0.357	0.481	0.010
214	MFG.L	SAL	(-28, 52, 21)	4.311	0.041	0.124	0.048
**215**	**ACG.L**	**SAL**	**(0, 30, 27)**	**10.397**	**0.002**	**0.030**	**0.109**
216	DCG.R	SAL	(5, 23, 37)	5.917	0.017	0.081	0.065
217	ACG.R	SAL	(10, 22, 27)	0.602	0.440	0.543	0.007
218	SFGdor.R	SAL	(31, 56, 14)	2.595	0.111	0.219	0.030
219	MFG.R	SAL	(26, 50, 27)	3.517	0.064	0.161	0.040
220	MFG.L	SAL	(-39, 51, 17)	2.275	0.135	0.251	0.026
**221**	**MFG.R**	**SAL**	**(2, -24, 30)**	**10.417**	**0.002**	**0.030**	**0.109**
222	THA.R	SUB	(6, -24, 0)	1.357	0.247	0.369	0.016
223	THA.L	SUB	(-2, -13, 12)	2.995	0.087	0.197	0.034
224	THA.L	SUB	(-10, -18, 7)	4.148	0.045	0.131	0.047
225	THA.R	SUB	(12, -17, 8)	3.627	0.060	0.154	0.041
226	THA.L	SUB	(-5, -28, -4)	5.800	0.018	0.083	0.064
227	PUT.L	SUB	(-22, 7, -5)	3.140	0.080	0.188	0.036
228	CAU.L	SUB	(-15, 4, 8)	5.270	0.024	0.095	0.058
229	PUT.R	SUB	(31, -14, 2)	0.086	0.769	0.806	0.001
230	PUT.R	SUB	(23, 10, 1)	1.099	0.298	0.425	0.013
231	PUT.R	SUB	(29, 1, 4)	0.261	0.611	0.683	0.003
232	PUT.L	SUB	(-31, -11, 0)	0.642	0.425	0.537	0.007
233	CAU.R	SUB	(15, 5, 7)	2.747	0.101	0.210	0.031
234	THA.R	SUB	(9, -4, 6)	6.896	0.010	0.066	0.075
235	STG.R	VAN	(54, -43, 22)	1.467	0.229	0.352	0.017
236	MTG.L	VAN	(-56, -50, 10)	4.164	0.044	0.131	0.047
237	STG.L	VAN	(-55, -40, 14)	5.996	0.016	0.081	0.066
238	STG.R	VAN	(52, -33, 8)	3.749	0.056	0.150	0.042
239	MTG.R	VAN	(51, -29, -4)	7.945	0.006	0.053	0.085
240	MTG.R	VAN	(56, -46, 11)	5.955	0.017	0.081	0.065
241	IFGtriang.R	VAN	(53, 33, 1)	0.018	0.893	0.918	<0.001
242	IFGtriang.L	VAN	(-49, 25, -1)	1.741	0.191	0.314	0.020
243	No	No	(-16, -65, -20)	1.480	0.227	0.351	0.017
244	No	No	(-32, -55, -25)	5.463	0.022	0.088	0.060
245	No	No	(22, -58, -23)	7.252	0.009	0.059	0.079
246	No	No	(1, -62, -18)	4.385	0.039	0.120	0.049
247	No	No	(33, -12, -34)	0.427	0.515	0.610	0.005
248	No	No	(-31, -10, -36)	2.990	0.087	0.197	0.034
249	No	No	(49, -3, -38)	2.197	0.142	0.255	0.025
250	No	No	(-50, -7, -39)	6.418	0.013	0.072	0.070
251	PCUN.R	DAN	(10, -62, 61)	0.388	0.535	0.619	0.005
252	MTG.L	DAN	(-52, -63, 5)	5.215	0.025	0.095	0.058
253	No	No	(-47, -51, -21)	0.126	0.724	0.767	0.001
254	No	No	(46, -47, -17)	4.703	0.033	0.108	0.052

255	IPL.R	MSN	(47， -30， 49)	0.795	0.375	0.494	0.009
256	SOG.R	DAN	(22， -65， 48)	1.255	0.266	0.390	0.015
257	MTG.R	DAN	(46， -59， 4)	3.923	0.051	0.144	0.044
258	SPG.R	DAN	(25， -58， 60)	5.941	0.017	0.081	0.065
**259**	**IPL.L**	**DAN**	**(-33，-46，47)**	**11.330**	**0.001**	**0.030**	**0.118**
260	SOG.L	DAN	(-27， -71， 37)	1.598	0.210	0.333	0.018
261	MFG.L	DAN	(-32， -1， 54)	0.843	0.361	0.484	0.010
262	ITG.L	DAN	(-42， -60， -9)	2.367	0.128	0.242	0.027
263	SPG.L	DAN	(-17， -59， 64)	2.949	0.090	0.200	0.034
**264**	**PreCG.R**	**DAN**	**(29，-5，54)**	**12.387**	**0.001**	**0.026**	**0.127**
